# Composition of Nonextractable Polyphenols from Sweet Cherry Pomace Determined by DART-Orbitrap-HRMS and Their *In Vitro* and *In Vivo* Potential Antioxidant, Antiaging, and Neuroprotective Activities

**DOI:** 10.1021/acs.jafc.2c03346

**Published:** 2022-06-22

**Authors:** Gloria Domínguez-Rodríguez, Daniel Ramón Vidal, Patricia Martorell, Merichel Plaza, María Luisa Marina

**Affiliations:** † Universidad de Alcalá, Departamento de Química Analítica, Química Física e Ingeniería Química, Facultad de Ciencias, Ctra. Madrid-Barcelona Km. 33.600, 28871 Alcalá de Henares, Madrid, Spain; ‡ Mendel University in Brno, Department of Chemistry and Biochemistry, Zemedelska 1, CZ-613 00 Brno, Czech Republic; § Archer Daniels Midland, Nutrition, Health&Wellness, Biopolis S.L. Parc Scientific Universitat de València, C/Catedrático Agustín Escardino Benlloch, 9, Paterna, 46980 Valencia, Spain; ∥ Universidad de Alcalá, Instituto de Investigación Química Andrés M. del Río (IQAR), Ctra. Madrid-Barcelona. Km. 33.600, 28871 Alcalá de Henares, Madrid, Spain

**Keywords:** nonextractable polyphenols, *Caenorhabditis elegans*, high-performance thin-layer chromatography, direct analysis in real-time high-resolution mass spectrometry, cherry pomace

## Abstract

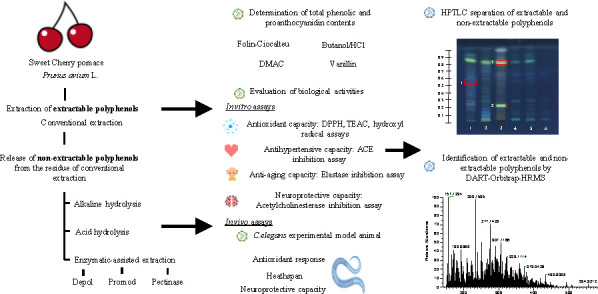

Sweet cherry pomace is an important source of phenolic compounds with beneficial health properties. As after the extraction of phenolic compounds, a phenolic fraction called nonextractable polyphenols (NEPs) remains usually retained in the extraction residue, alkaline and acid hydrolyses and enzymatic-assisted extraction (EAE) were carried out in this work to recover NEPs from the residue of conventional extraction from sweet cherry pomace. *In vitro* and *in vivo* evaluation of the antioxidant, antihypertensive, antiaging, and neuroprotective capacities employing *Caenorhabditis elegans* was achieved for the first time. Extractable phenolic compounds and NEPs were separated and identified by families by high-performance thin-layer chromatography (HPTLC) with UV/Vis detection. A total of 39 phenolic compounds were tentatively identified in all extracts by direct analysis in real-time high-resolution mass spectrometry (DART-Orbitrap-HRMS). EAE extracts presented the highest *in vitro* and *in vivo* antioxidant capacity as well as the highest *in vivo* antiaging and neuroprotective capacities. These results showed that NEPs with interesting biological properties are retained in the extraction residue, being usually underestimated and discarded.

## 1 Introduction

Oxidative stress is characterized by the uncontrolled formation of reactive oxygen species (ROS) and an imbalance in the biological system’s capacity to repair the cellular damage that increases with aging. The increase in ROS levels induces lipid peroxidation in cell membranes and initiates neuronal dysfunction and neuronal death causing different diseases such as Alzheimer’s or Parkinson’s diseases, among other pathological situations.^1,2^ Alzheimer’s disease is characterized by an accumulation of intraneuronal filaments formed by the microtubule-associated protein tau, acetylcholine degradation, and aggregation of amyloid-β protein (a pro-inflammatory agent) in the brain parenchyma and cerebral blood vessels, which are associated with the loss of neurons and their functions, a process increased in the brain with aging.^3,4^ Inhibitors of the acetylcholinesterase enzyme, such as tacrine or galantamine, are the most used medications for the treatment of Alzheimer’s disease delaying the degradation of released acetylcholine by enhancing cholinergic neurotransmission. Also, different researchers have demonstrated that patients receiving anti-inflammatory therapies have decreased risk for developing Alzheimer’s disease, and antihypertensive medications are used to increase blood flow in patients with Alzheimer’s disease.^5−8^ However, current drugs employed to mitigate some of the symptoms of Alzheimer’s disease cause undesirable secondary effects (nausea, diarrhea, insomnia, etc.).^9^ For this reason, there is growing interest in finding alternative treatments from natural sources to prevent damage to cells by ROS and acetylcholine degradation. In this sense, fruits are recognized for their high concentrations of natural antioxidants and anti-inflammatory and antihypertensive compounds, such as phenolic compounds.^10,11^ These compounds have demonstrated important antioxidant, anti-inflammatory, and antihypertensive effects, playing a relevant role in the prevention of neurological pathologies like Alzheimer’s disease.^3,9,12−16^ Phenolic compounds are mainly concentrated in fruit peels, which causes their recovery in industry processing to be low because peels are considered waste material.^17,18^ In particular, the processing of sweet cherries (*Prunus avium* L.) generates a high amount of byproducts since the global production of this fruit is about 2.2 million tons.^19^ Interestingly, several researchers have demonstrated that sweet cherry pomace could be a valuable source of bioactive compounds.^20,21^

Sweet cherry has been described as a source of phenolic compounds with antioxidant capacity, which include hydroxycinnamates, anthocyanins, catechins, and flavonols.^22,23^ In addition, proanthocyanidins and flavonoid compounds found in sweet cherries have been shown to reduce risk of Alzheimer’s disease by reducing oxidant stress and the production of β-amyloid, protecting neuronal cells.^22^ Regarding sweet cherry pomace, Dominguez-Rodriguez et al. (2021) described extracts with phenolic compounds with antioxidant and antihypertensive capacities.^21^ Also, antioxidant extractable polyphenols were obtained from sweet cherry pomace by pulsed electric fields.^24^ However, the studies about the characterization and analysis of the bioactivity of phenolic compounds from sweet cherry pomace are very limited.^21^

Usually, phenolic compounds are obtained from foods by aqueous and organic solvents.^13^ Nevertheless, the analysis of phenolic compounds from different matrices omits other phenolic compounds that are retained in the residue of the food matrix after aqueous–organic extraction.^26^ This underestimated fraction corresponds to nonextractable polyphenols (NEPs), which are low molecular weight polyphenols called hydrolyzable polyphenols associated with macromolecules such as proteins or dietary fiber or high molecular weight polyphenols, which are mostly nonextractable proanthocyanidins.^26^ These compounds interact with the food matrix by hydrogen and covalent bonds or hydrophobic interactions or even extractable polyphenols could be associated with NEPs.^26^

To recover NEPs from the aqueous–organic extraction residue, alkaline hydrolysis, acid hydrolysis, or enzymatic-assisted extraction (EAE) methods are employed. Alkaline and acid hydrolyses are the most used extraction methodologies to obtain NEPs.^27^ Nevertheless, in these extraction techniques, some phenolic compounds are not stable to the high and low pH.^27,28^ For these reasons, EAE has been reported as a sustainable treatment where the residue of the extraction does not receive any excessive pH alteration, being more selective and efficient to release NEPs from the food matrix than acid and alkaline hydrolyses.^28^ Casein protease, esterase, endogalacturonase, cellulase, pectinase, tannase, and α-amylase enzymes have been employed to release NEPs from the residue.^29−31^

Regarding the characterization of NEPs, preparative high-performance liquid chromatography in the reversed-phase mode (RP-HPLC), high-speed counter-current chromatography (HSCCC), or normal phase HPLC (NP-HPLC) coupled to UV/Vis detectors, electrospray ionization mass spectrometry (ESI-MS), or matrix-assisted laser desorption ionization/time-of-flight mass spectrometry (MALDI-TOF-MS) have been used.^32−35^ Nevertheless, NEPs from sweet cherry pomace have not been characterized to date. High-performance thin-layer chromatography (HPTLC) could be an interesting alternative to separate NEPs from different samples in a unique analysis to be subsequently characterized by direct analysis in real-time and acquiring high-resolution mass spectra (DART-HRMS) with high accuracy and precision isotopic abundance measurements with Orbitrap analyzer.^36^

The determination of the phenolic composition of the extracts is crucial to obtain a broad knowledge about what type of phenolic compounds exert the *in vitro* and *in vivo* beneficial properties. Even though the beneficial health properties exhibited by NEPs from sweet cherry pomace have been reported, their toxicological effect has not been evaluated. Thus, before extracts rich in NEPs can be included in clinical studies or used as ingredients in any product, their toxicity must be tested because some of their compounds may be potentially toxic or carcinogenic.^37^

The traditional animal model for the *in vivo* study of the bioactivity of phenolic compounds is a rodent, primarily rats and mice. However, *Caenorhabditis elegans* (*C. elegans*) is an attractive animal model extensively used for research involving aging and neurodegenerative diseases for which research approval by Animal Care and Use Committees is not required.^38^ This nematode, length of 1 mm, has 65–80% of genes associated with humans.^39^ Results obtained with this nematode are consistent with those from other animals such as rodents enabling subsequent preclinical and clinical assays to be more focused.^40^ The antiaging capacity of phenolic compounds of juice from sour cherries has been studied through *C. elegans*, but to our knowledge, a study about the *in vivo* bioactivity of NEPs obtained from sweet cherry pomace has not been reported in the literature.^41^

Therefore, the main aim of this work was to revalorize sweet cherry pomace evaluating the efficiency of the extraction of NEPs by acid, alkaline, and EAE methods (obtaining three extracts, one with high bioactivity, another with high polyphenol content, and another with high antioxidant polyphenol content) to be compared with extractable polyphenol fraction estimating the contribution to the *in vitro* and *in vivo* antioxidant, antihypertensive, antiaging, and neuroprotective capacities. *In vivo* assays were carried out using *C. elegans* as an experimental animal model. Additionally, the characterization of the extracts was achieved by HPTLC–UV/Vis to classify the separated phenolic compounds by families and by DART-Orbitrap-HRMS to obtain a rapid and tentative phenolic fingerprint of the extracts.

## 2 Materials and Methods

### 2.1 Chemicals and Samples

Ethanol, acetone (99.9%), formic acid (98–100%), and hydrochloric acid (37%) of HPLC grade were supplied by Scharlab Chemie (Barcelona, Spain), and methanol (99.99%), butanol, and sulfuric acid were supplied by Fisher Scientific (Leicestershire, UK). Gallic acid, epicatechin, vanillin, sodium carbonate, sodium hydroxide, sodium chloride, Folin–Ciocalteu reagent, 4-dimethylaminocinnamaldehyde (DMAC), 6-hydroxy-2,5,7,8-tetramethylchromane-2-carboxylic acid (Trolox), potassium persulfate, 2,2′-azinobis(3-ethylbenzothiazoline-6-sulfonic acid) diammonium salt (ABTS), 2,2-diphenyl-1-picrylhydrazyl (DPPH^•^), iron(III) chloride, ethanolamine, 1,10-phenanthroline, captopril, *N*-succinyl-Ala-Ala-Ala-*p*-nitroanilide, elastase from porcine pancreas, 5,5-dithiobis[2-nitrobenzoic acid], acetylcholinesterase (AChE), acetylthiocholine iodide (ATCI), galantamine, trifluoroacetic acid (TFA), angiotensin-converting enzyme (ACE) from rabbit lung, hippuryl-histidyl-leucine (HHL), and 2-[4-(2-hydroxyethyl)-1-piperazinyl]-ethanesulfonic acid (HEPES) were obtained from Sigma-Aldrich (Saint Louis, MO, USA). Dipotassium hydrogen phosphate and sodium dihydrogen phosphate dihydrate were supplied from Merck (Darmstadt, Germany). Ethyl acetate, toluene, and sodium sulfate were provided by Penta (Chrudim, Czech Republic).

Ultrapure water (18.2 MΩ/cm) was generated with a Millipore system (Millipore, Billerica, MA, USA). Depol 740 L, Promod 439 L, and Pectinase 62 L enzymes were kindly donated by the company Biocatalysts Limited (Cardiff, UK).

Sweet cherries belonging to *Prunus avium* L., Early Lory variety, Rosaceae family were collected in 2019 from La Almunia de Doña Godina (Zaragoza, Spain). To obtain the fruit pomace, the fruits were washed, destemmed, destoned, and pressed manually. Finally, pomace was ground in a commercial blender and stored at −20 °C until analysis.

### 2.2 Conventional Extraction of Extractable Polyphenols

Extractable polyphenols were obtained based on the method in a previous study performed by Dominguez-Rodriiguez et al. (2021).^21^ Briefly, 20 mL of methanol/water (50:50 v/v) acidified with 2 M HCl (pH 2) was added to 15 g of cherry pomace and incubated for 1 h at room temperature with shaking. After that, the extract was centrifuged for 10 min at 2100*g* to obtain the supernatant. The extraction residue was mixed with 20 mL of acetone/water (70:30, v/v), shaken for 1 h at room temperature, and centrifuged at 2100*g* for 10 min. Finally, both supernatants were combined and stored at −20 °C until analysis, and the extraction residue was stored to be used for the extraction of NEPs. Samples were prepared in triplicate.

### 2.3 Extraction of Nonextractable Polyphenols

#### 2.3.1 Enzymatic-Assisted Extraction

EAE was carried out according to the optimal extraction conditions obtained in the experimental designs performed by our research group to extract high content of bioactive NEPs (HBN extract) from sweet cherry pomace employing three different enzymes (Depol 740L (Depol) with β-glucanase activity, Promod 439L (Promod) with protease and polygalacturonase activities, and Pectinase 62L (Pectinase) with pectin lyase activity).^21^ In this previous work, a Box–Behnken experimental design was used for each enzyme to determine the influence of enzyme concentration, pH, extraction time, and temperature in the NEP extraction from the extraction residue of sweet cherry pomace. Extractions were achieved using phosphate buffer (100 mM) as extraction solvent, 0.38 g of sample/mL, and enzyme concentrations of 90 μL of Depol enzyme, 140 μL of Promod enzyme, and 2 μL of Pectinase per gram of sample according to the method of Dominguez-Rodriguez et al. (2021).^21^

The design consisted of 29 randomized runs for each enzyme with three levels and five central points. The response variables were total phenolic content (Folin–Ciocalteu method), total proanthocyanidin content (DMAC, vanillin, and butanol/HCl assays), antioxidant capacity (DPPH, Trolox equivalent antioxidant capacity (TEAC), and the capacity to inhibit the hydroxyl radical assays), and antihypertensive capacity (ACE inhibition assay). The evaluation of the adequacy of fitted models settled between parameters to optimize and the different responses was carried out by analysis of variance (ANOVA).

Using this experimental design, the theoretical optimal extraction conditions to obtain extracts with high bioactivity (HB extract) and high content of phenolic compounds and PAs (TPA extract) from sweet cherry pomace were also calculated by using graphical and numerical methods based on the criteria of the desirability function and the response surface plots. Table S1 shows the theoretical optimal extraction conditions to obtain HBN, HB, and TPA extracts of each enzyme obtained from the experimental designs performed in a previous study.^21^ The EAEs with Depol, Promod, and Pectinase enzymes were performed in triplicate under the theoretical optimal extraction conditions obtained from the experimental design to corroborate the study.

#### 2.3.2 Acid and Alkaline Hydrolyses

Acid hydrolysis as described by Hartzfeld et al. (2002) was employed to extract NEPs from the residue of cherry pomace with some modifications.^42^ Briefly, 0.38 g of extraction residue was mixed with 1 mL of methanol/H_2_SO_4_ (90:10, vol %) by shaking for 20 h at 85 °C in a thermoreactor (Spectroquant TR420, Merck, Germany). Then, the extracts were submitted to centrifugation at 3000*g* for 10 min, and the supernatants were collected. Subsequently, extracts were washed twice with distilled water, and the final volume was adjusted to 2 mL. Finally, 200 μL of ethanolamine was added with agitation, and pH was adjusted to 5.5.

On the other hand, alkaline extraction was carried out as previously reported by Arranz and Saura-Calixto (2010) for the extraction of NEPs.^43^ Extraction residue (9.38 g) was mixed with 25 mL of 2 M NaOH for 4 h at room temperature. In order to neutralize the mixture, an appropriate amount of hydrochloric acid was added (pH 3.0). Acid and alkaline hydrolyses were conducted in triplicate.

### 2.4 Total Phenolic and Proanthocyanidin Contents

Total phenolic content was determined following the Folin–Ciocalteu (FC) method based on the work by Kosar et al. (2005), and proanthocyanidin content was determined according to DMAC, vanillin, and butanol/HCl assays used by Montero et al. (2013), Gu et al. (2008), and Pérez-Jiménez et al. (2009), respectively, employing a Cary 8454 UV–vis spectrophotometer (Agilent Technologies, Palo Alto, CA, USA).^29,44−46^ The results were expressed as milligrams of epicatechin per 100 g of sample.

### 2.5 High-Performance Thin-Layer Chromatography Separation of Extractable Polyphenols and NEPs

The extracts were preconcentrated with ethyl acetate to obtain greater band intensity on the TLC plate and greater signal intensity in the DART-Orbitrap-HRMS analysis according to the method of Dominguez-Rodriguez et al. (2021).^47^ The liquid was evaporated, and the residue was reconstituted in 200 μL of methanol to be injected into the HPTLC system and for the analysis by DART-Orbitrap-HRMS.

Six samples were applied in a volume of 10 μL using a semiautomatic applicator (CAMAG LINOMAT 5, Muttenz, Switzerland) with an HPTLC syringe of 100 μL (Hamilton, Bonaduz, Switzerland) employing 6 mm of band length with a distance between tracks of 15.4 mm on normal phase (NP) HPTLC plates (HPTLC Silica Gel 60 F254 Plates 20 cm × 10 cm). A CAMAG (Muttenz, Switzerland) instrument was used to separate extractable polyphenols and NEPs from sweet cherry pomace extracts.

Chromatography separation was performed following the method described by Dominguez-Rodriguez et al. (2021) where ethyl acetate–toluene–formic acid–methanol (6:6:1.6:0.4, v/v/v/v) was employed as the mobile phase.^47^ Development took 40 min, and the plate was removed from the chamber and dried in a TLC heater at 60 °C for 15 min.

Spectral analysis was performed in a TLC Scanner (CAMAG) from 200 to 800 nm obtaining the retention factors (*R_f_*), peak areas in absorbance units (AU), and wavelengths at absorption maximum in nanometers of separated substances. Then, the developed plate was sprayed in a derivatizer (Camag, Muttenz, Switzerland) using 2 mL of 10% H_2_SO_4_ in methanol and dried using the TLC heater at 60 °C. Before and after derivatization, digital pictures were taken under 254 and 366 nm UV light and white light above the plate using a TLC visualizer (CAMAG, Muttenz, Switzerland) equipped with a 12 bi-bit charge-coupled device (CCD) digital camera.

### 2.6 DART-Orbitrap-HRMS Analysis

Extractable polyphenols (EPPs) obtained by conventional extraction and NEPs recovered by alkaline hydrolysis and EAE with Promod, Depol, and Pectinase enzymes from sweet cherry pomace were tentatively identified by DART-Orbitrap-MS. DART ionization was performed in a DART-Standardized Voltage and Pressure Adjustable (SVPA) device using the method described by Falk et al. (2018).^48^ The DART ion source worked in negative and positive ionization modes with helium ionizing gas at 0.55 MPa pressure, 350 °C beam temperature, and 350 V grid electrode voltage. High-resolution mass spectral (HRMS) measurements were performed on an Orbitrap mass spectrometer (Thermo Fischer Scientific, Bremen, Germany) coupled to an ion source through an interface evacuated with a diaphragm pump. The linear ion trap mass spectrometer settings were as follows: capillary voltage 50 V; tube lens voltage 100 V; skimmer voltage 18 V; capillary temperature 300 °C.

To perform data acquisition and processing, the Xcalibur software (Thermo Fischer Scientific, Germany) with DART web-based module was employed. The acquisition rate was set to 2 spectra per second providing resolution of 120 000 full width at half-maximum (fwhm) at *m*/*z* 200.

Liquid extracts were pipetted (10 μL) onto DART-QuickStrip plates for the analysis while residues of the extractions (solid sample) were analyzed employing tweezers.

### 2.7 Antioxidant Capacity Determination

The DPPH radical scavenging capacity was determined using the method described by Brand-Williams et al. (1995).^49^ The concentration to decrease the initial DPPH concentration by 50% (EC_50_) was calculated by plotting the percentage of remaining DPPH on a graph against the sample concentration using a calibration curve of DPPH. Thereby, a greater EC_50_ implies less antioxidant capacity in extracts.

Also, the TEAC assay was applied following the method of Re et al. (1999).^50^ Trolox was used as the reference standard to express the results as TEAC (Trolox equivalent antioxidant capacity) values (mmol Trolox/g extract) employing a standard curve. The TEAC values were obtained from four different concentrations of each extract giving a linear response between 20% and 80% compared with the initial absorbance. Analyses were done in triplicate for each extract.

On the other hand, a hydroxyl radical assay based on the protocol of Dominguez-Rodriguez et al. (2021) was employed to determine the capacity to inhibit the formation of hydroxyl radicals.^21^ The results were expressed as % inhibition of hydroxyl radical formation.

### 2.8 Antihypertensive Capacity

Angiotensin-converting enzyme (ACE) inhibition was used to determine antihypertensive capacity from cherry pomace following the method of Geng et al. (2010) with some modifications.^21,51^ Results were expressed as a percentage of ACE inhibition using the following equation:
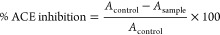
where *A*_control_ is the area under the peak of HA (hippuric acid) in the control and *A*_sample_ is the area under the peak of HA in the sample.

Moreover, the concentration required for the 50% inhibition of ACE activity (IC_50_) was calculated for the extracts obtained under the optimal conditions by EAE and the extracts performed by conventional extraction and acid and alkaline hydrolysis.

### 2.9 Elastase Inhibition Activity

Elastase inhibition activity assay based on Azmi et al. (2014) with some modifications was employed to determine the antiaging capacity of the extracts.^52^ Briefly, 100 μL of 0.2 mM Tris-HCl buffer (pH 8.0), 25 μL of 10 mM *N*-succinyl-Ala-Ala-Ala-*p*-nitroanilide dissolved in the Tris-HCl buffer, and 50 μL of extract were mixed. After incubation for 15 min at 25 °C, absorbance was measured at 410 nm. Then, 25 μL of 0.3 units/mL elastase was added and incubated for another 15 min at 25 °C and the absorbance was read at 410 nm in a Cary 8454 UV–Vis spectrophotometer (Agilent Technologies, Palo Alto, CA, USA). Epicatechin (0.7 mg/mL) was used as a positive control. The results were expressed as % of elastase inhibition activity employing the following equation:
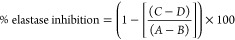
where *C* is the absorbance of the extract after incubation with the enzyme, *D* is the absorbance of the extract after incubation without enzyme, *A* is the absorbance of the control after incubation with enzyme and *B* is the absorbance of the control after incubation without enzyme.

### 2.10 Acetylcholinesterase Inhibition Activity Assay

Acetylcholinesterase (AChE) inhibition activity was measured using Ellman’s method as described by Mathew and Subramanian (2014) with some modifications.^53^ In brief, 100 μL of 3 mM of DTNB (5,5-dithiobis[2-nitrobenzoic acid]) dissolved in 50 mM Tris-HCl buffer (pH 8.0) containing 0.1 M NaCl and 0.02 M MgCl_2_, 20 μL of 0.26 U/mL AChE dissolved in 0.1% BSA (bovine serum albumin) in buffer, 640 μL of buffer, and 20 μL of the extract were mixed. After incubation for 15 min at 25 °C, absorbance was measured at 412 nm in a Cary 8454 UV–Vis spectrophotometer (Agilent Technologies), which was treated as the control. Then, the enzymatic reaction was started by the addition of 15 mM ATCI (acetylthiocholine iodide) dissolved in water, and the absorbance was read at 412 nm until the reaction completed (45 min). Galantamine (100 μM) was used as the positive control. The results were expressed as % inhibition of AChE employing the following equation:
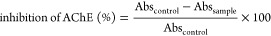
where Abs_control_ is the absorbance containing all reagents except ATCI and Abs_sample_ is the absorbance of the solution prepared after completing the enzymatic reaction with ATCI.

### 2.11 *Caenorhabditis elegans* Strains and Maintenance

*C. elegans* strain N2, var. Bristol (wild-type), and the transgenic strain CL4176 (smg-1ts [pAF29(myo-3/Aβ1–42/let UTR)+pRF4(rol-6(su10069))]) were obtained from the *Caenorhabditis* Genetics Center at the University of Minnesota. N2 worms were maintained at 20 °C, while strain CL4176 was maintained at 16 °C, both on Nematode Growth Medium (NGM) plates (agar 17.5 g/L, sodium chloride 3.0 g/L, peptone 2.5 g/L, and cholesterol 0.005 g/L) with *Escherichia coli* strain OP50 as the normal diet for nematodes for all experimental assays.

#### 2.11.1 Antioxidant Response in *C. elegansIn Vivo* Assay

The wild-type strain N2 of *C. elegans* (var. Bristol) was used as an *in vivo* model to evaluate the antioxidant capacity of the extracts. The experiment was performed as described by Martorell et al. (2011).^54^ To obtain age-synchronized nematodes, eggs were isolated from gravid adults and hatched overnight in NGM plates. NGM plates were supplemented with different sweet cherry extracts at two different concentrations (extracts were diluted in 5% DMSO at 100 and 400 μg/mL) using NGM medium as control and vitamin C (10 μg/mL) as positive control. Worms (50 worms/fed condition) were incubated at 20 °C under these conditions. Once the adult phase was achieved (5 days), nematodes were transferred to a basal medium containing 2 mM H_2_O_2_ to induce oxidative stress. After 5 h of incubation, the total number of worms that survived the treatment was counted. A test was conducted as a screening due to the high reproducibility of the assay (one assay was performed for each extract).

#### 2.11.2 Health Span in *C. elegansIn Vivo* Assay

Aging is characterized by a loss of body movement. Like humans, *C. elegans* lose movement with aging, and they can move only their heads. For this reason, the mobility of nematodes was evaluated as an aging-related parameter. An automated system based on artificial vision was used to score the activity of worms under the different treatments during the first 4 days of adulthood.

Age-synchronized nematodes of wild-type strain N2 were used for the antiaging assay using 96-well plates with solid medium (NGM). To find the optimum extract dose, nematodes were cultured using three different amounts of extracts (10, 20, and 30 μL for all the extracts, except for the acid extract, tested at amounts of 2.5, 5.0, and 10.0 μL to avoid a detrimental effect on *C. elegans* due to the pH). A control condition without extract was included.

Mobility was tracked during 4 days at 20 °C, and the fold change of worm mobility (activity treatment/activity control) was estimated each day to normalize data.

#### 2.11.3 Neuroprotective Capacity of Extracts in *C. elegansIn Vivo* Assay

The evaluation of the neuroprotective capacity of the extracts on *C. elegans* was carried out with the transgenic *C. elegans* strain CL4176, which can produce the neurotoxic peptide amyloid β-peptide in either neurons or body wall muscle.^55^ The paralysis produced by the expression of the human amyloid β-peptide in the *C. elegans* strain was measured. Age-synchronized worms were cultured in NGM as control and NGM supplemented with each sample at three different volumes in the plate (100, 200, and 300 μL excepting acid extract, which was added in a concentration of 10, 25, and 50 μL to avoid detrimental effects in the development of nematodes) at 16 °C until L3 stage (larval stage 3 that corresponds to 9 h after fertilization). At this time, transgene expression was induced in nematodes by up-shifting the temperature from 16 to 25 °C. Worms were maintained at 25 °C until 100% of worms became paralyzed. Paralysis in induced worms was compared with noninduced worms (maintained at 16 °C until the end of the paralysis assay).

*Ginkgo biloba* EGb761 (100 μg/mL) was used as positive control. Assays were performed in duplicate.

### 2.12 Cell Culture, Treatments, and Cell Viability

All the cells used in this study were obtained from the American Type Culture Collection ATCC (Rockwell, MD, USA) and cultured in an incubator at 37 °C with 5% CO_2_ saturation and 95% humidity in their culture medium.

Hepatocarcinoma HepG2, primary dermal fibroblast HFF-1, and human ovarian cancer SKOV3 cell lines were maintained in Dulbecco’s modified Eagle’s medium (DMEM); colon adenocarcinoma HT-29 cells were maintained in MacCoy’s 5A medium. All cell lines were supplemented with 10% fetal bovine serum and antibiotics.

The cell lines mentioned above were used to determine the *in vitro* cytotoxic effect of conventional, alkaline, acid, and EAE extracts at different concentrations (0.380, 0.285, 0.095, 0.038, 0.019, and 0.0095 mg/mL extract) on the cell viability by the MTT [3-(4,5-dimethylthiazole-2-yl)2,5-diphenyltetrazolium bromide] assay as described by Hernández-Corroto et al. (2018) with some modifications.^56^ Briefly, cells were seeded at a density of 5000 cells/well in a 96-well plate and incubated with 10 μL of extract for 24 h. Afterward, 10 μL of MTT stock solution at 5 mg/mL in phosphate buffer was added to each well and incubated for 6 h. Then, the culture medium was removed and formazan crystals were dissolved with 100 μL of DMSO. Finally, absorbance was measured at 570 nm. Results were expressed as a percentage of cell viability after 24 h concerning the control according to the following formula:
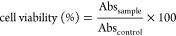
where Abs_sample_ and Abs_control_ are the absorbances of remaining formazan when cells were treated with the extracts and extraction solvent, respectively.

### 2.13 Statistical Analysis

The program Statgraphics Centurion XVII (Statistical Graphics Corp., USA) was used for statistical analysis. Analysis of variance (ANOVA) by Fisher’s exact test to discriminate on the least significant difference LSD (*p* ≤ 0.05) was used to compare differences in total phenolic content and total proanthocyanidin content of cherry pomace extracts as well as in antioxidant, antihypertensive, antiaging, and neuroprotective capacities for *in vitro* assays. To compare the effect on paralysis protection of each sample versus control-fed nematodes, one-way ANOVA and Tukey’s multiple comparison tests were applied. All statistical analyses for *in vivo* assays were performed in GraphPad Prism 9 statistical software package.

## 3 Results and Discussion

In this work, EAE and alkaline and acid hydrolyses were employed to release NEPs from conventional extraction residue of cherry pomace to determine the total phenolic and proanthocyanidin contents and evaluate the *in vitro* and *in vivo* antioxidant, antihypertensive, antiaging, and neuroprotective capacities of these extracts.

The optimal extraction conditions to obtain HBN, TPA, and HB extracts by EAE (see Table S1) from sweet cherry pomace were obtained from a Box–Behnken experimental design previously elaborated by our research group to determine the influence of enzyme concentration, time, temperature, and pH on the total phenolic and proanthocyanidin contents, antioxidant capacity, and antihypertensive capacity.^21^ HBN theoretical values obtained from the experimental design were experimentally verified by Dominguez-Rodriguez et al. (2021).^21^ In addition, theoretical optimal values obtained for total phenolic content (TPC), PA contents, and antioxidant and antihypertensive capacities from the optimal extraction conditions to obtain TPA and HB extracts from the residue of conventional extraction of cherry pomace were submitted for validation through an experimental analysis in this study.

Table S2 shows the theoretical optimal values from FC, DMAC, vanillin, and butanol/HCl assays that should be obtained under the optimal extraction conditions for Depol, Promod, and Pectinase enzymes to obtain TPA extracts along with a range of values within which the experimental values must be included. Experimental values were lower than the theoretical ones except for Promod enzyme, for which experimental value was within the range of the predictive model in DMAC assay (see Table S2). The experimental analysis was carried out with sweet cherries harvested in 2019, while theoretical values were obtained from sweet cherries harvested in 2018. The low experimental results obtained in this analysis compared with theoretical results may be because the cherries were harvested in different years with different weather conditions that may have varied the content of phenolic and proanthocyanidin compounds as well as their biological activity.

On the other hand, experimental values of DPPH and TEAC assays for HB extracts were within the range of the predictive model for each enzyme (see Table S3). However, experimental antioxidant values from hydroxyl radical assay were lower for Promod and Depol enzymes and higher for Pectinase enzyme than the theoretical ones.

In general, the predictive model from experimental design allows obtaining a good prediction for the antioxidant capacity using DPPH and TEAC assays to obtain HB extracts. However, this predictive model did not allow a good prediction of the TPC and PA values for TPA extracts.

### 3.1 Determination of the Total Phenolic and Proanthocyanidin Contents

Table 1 shows the TPC values of the extracts obtained by conventional extraction, alkaline and acid hydrolyses, and EAE from sweet cherry pomace. As it can be seen, results were statistically different (*p* ≤ 0.05) among extraction techniques.

**Table 1:** Total Phenolic Content (TPC) and Total Proanthocyanidin Content (DMAC, Vanillin, and Butanol/HCl Assays) Obtained by Different Extraction Methods from Cherry Pomace⊗

sample	TPC (mg GAE/100 g sample)	DMAC (mg epicat/100 g sample)	vanillin (mg epicat/100 g sample)	butanol/HCl (mg epicat/100 g sample)
conventional	8.30 ± 0.05^k^	0.0121 ± 0.0009^d^	2.9 ± 0.2^l^	25 ± 4^b^
acid	179.4 ± 0.1^a^	0.049 ± 0.003^b^	34.35 ± 0.07^a^	30.7 ± 0.2^a^
alkaline	136.9 ± 0.2^l^	0.027 ± 0.006^c^	9.60 ± 0.07^h^	9.2 ± 0.4^d^
Pectinase HBN	84.94 ± 0.03^f^	0.0298 ± 0.0008^c^	12.04 ± 0.01^d^	13.5 ± 0.5^c^
Pectinase TPA	62.88 ± 0.09^i^	0.016 ± 0.007^d^	9.39 ± 0.01^i^	16 ± 3^d^
Pectinase HB	48.13 ± 0.07^h^	0.05 ± 0.01^b^	8.97 ± 0.02^j^	9.5 ± 0.4^d^
Promod HBN	100.0 ± 0.4^e^	0.046 ± 0.001^b^	15.29 ± 0.02^d^	14 ± 1^c^
Promod TPA	72.76 ± 0.06^g^	0.01 ± 0.002^d^	17.262 ± 0.007^c^	10.3 ± 0.5^c^
Promod HB	70.84 ± 0.05^h^	0.0476 ± 0.0002^b^	28.675 ± 0.007^b^	9.4 ± 0.2^d^
Depol HBN	139.08 ± 0.04^b^	0.017 ± 0.002^d^	13.78 ± 0.03^e^	9.2 ± 0.3^d^
Depol TPA	137.84 ± 0.04^c^	0.0005 ± 0.0002^e^	6.66 ± 0.02^k^	9.4 ± 0.4^d^
Depol HB	109.32 ± 0.04^d^	0.063 ± 0.004^a^	9.97 ± 0.02^g^	13.7 ± 0.2^c^

⊗ Letters (a, b, c, d, e, f, g, h, i, j, k, l) show the significant differences among extraction methods of NEPs (*p* ≤ 0.05).

The richest extract in terms of TPC was achieved by acid hydrolysis followed by EAE with Depol enzyme from HBN and TPA extracts. The high phenolic content in acid hydrolysis could be due to the low pH employed in the treatment that allowed release of NEPs and other compounds trapped in the cores or conjugated to cell walls with macromolecules.^57^ Additionally, other reducing compounds different from phenolic compounds could be released from the cell wall of the extraction residue interfering in the measurement by the FC method and thus overestimating the TPC. By contrast, conventional extract presented the lowest TPC content observing that a high amount of phenolic compounds were retained in the extraction residue. Regarding EAE, HBN extracts showed higher TPC values than TPA extracts using the three enzymes. This result suggests that HBN extract presented other reducing agents with antioxidant capacity different from phenolic compounds compared with TPA extract because it was optimized to obtain high phenolic and proanthocyanidin contents and high antioxidant capacity.

On the other hand, Table 1 shows statistical differences (*p* ≤ 0.05) among the extraction methods employed to obtain NEPs using three different assays to measure PAs. Depol HB extract showed the highest PA content in DMAC assay. By contrast, Depol TPA extract showed the lowest PA content.

Regarding vanillin assay, acid hydrolysis was the most effective to extract PAs, while conventional extraction showed the lowest PA content. Acid hydrolysis also showed the highest PA content in the butanol/HCl assay. Alkaline hydrolysis showed the lowest PA content and did not show statistical differences with Pectinase HB, Promod HB, Pectinase TPA, Depol HB, and Depol HBN extracts (Table 1).

In the DMAC assay, the reagent reacts specifically with compounds with meta-oriented di- or trihydroxy phenols, as are found in PAs, while in the vanillin assay, the aldehyde group reacts with PAs but also with other flavonoids.^58^ To determine PA content with the vanillin assay, the absorbance is measured at 510 nm, absorbing in the same region as anthocyanins and overestimating the PA content. For this reason, the DMAC assay is preferable to measure PA content to the vanillin assay because it is more specific.^25,58^ These methods are not comparable to measure polymeric polyphenols because they are more specific for monomeric compounds being used as reference standards, while the butanol/HCl assay is more specific for polymeric compounds.^25,58^ In this sense, Table 1 shows that acid and EAE with Depol enzyme were the most effective treatments to obtain monomeric NEPs from conventional extraction residue, while acid treatment was the most efficient to obtain polymeric compounds.

### 3.2 Chromatographic Separation by HPTLC-UV/Vis of EPPs and NEPs Obtained from Conventional Extraction, Acid and Alkaline Hydrolyses, and EAE Using HBN Methodology

The extracts of EPPs obtained by conventional extraction as well as the NEP extracts recovered by acid and alkaline hydrolyses and EAE with Depol, Promod, and Pectinase enzymes were analyzed by HPTLC to determine their phenolic profiles. In addition, the extracts obtained by EAE under the optimal extraction conditions to obtain HBN extracts were chosen as the most representative extracts to characterize their NEP profiles because under these extraction conditions it is possible to release higher content of bioactive phenolic compounds and PAs.

**Figure 1:**
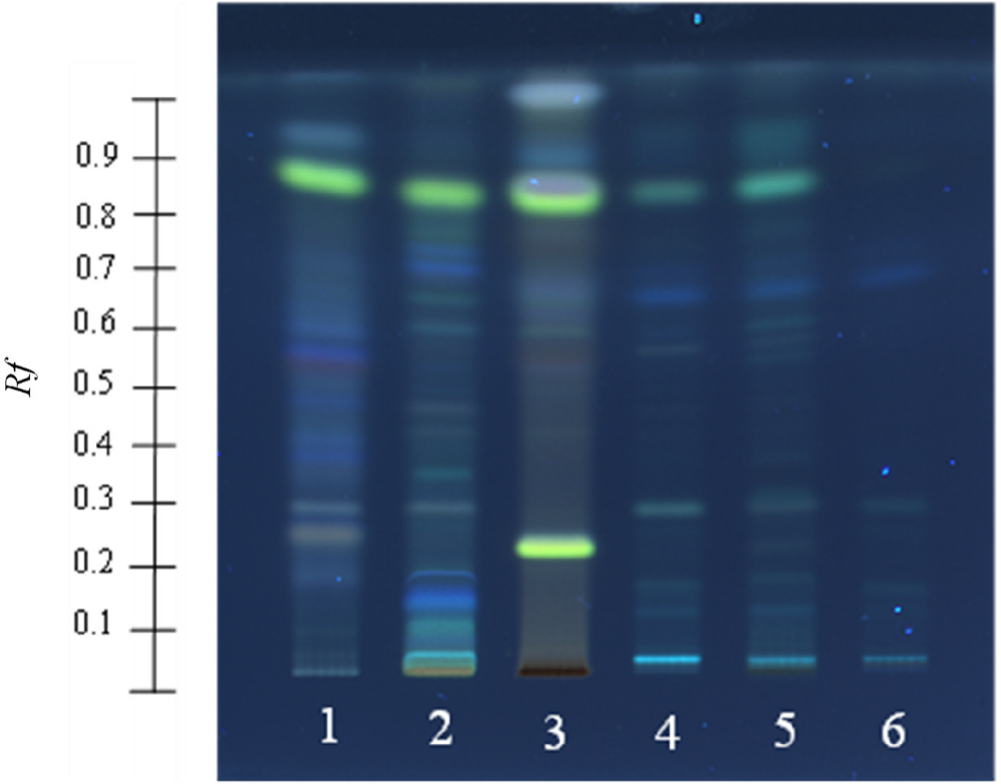
TLC visualization at 254 nm of separated bands of conventional extract and nonextractable polyphenol hydrolysates from sweet cherry pomace after derivatization (lane 1, conventional extraction; lane 2, alkaline hydrolysis; lane 3, acid hydrolysis; lane 4, EAE with Depol enzyme; lane 5, EAE with Promod enzyme; lane 6, EAE with Pectinase enzyme) by HPTLC using ethyl acetate–toluene–formic acid–methanol (6:6:1.6:0.4, v/v/v/v) as mobile phase.

Figure 1 shows the TLC plate visualization at 254 nm after the postchromatographic reaction through the addition of 10% of sulfuric acid in methanol to provide fluorescence for band visualization for the subsequent isolation and identification of compounds. The spot colors of the separated bands on the TLC plate detected by UV–vis light allowed us to group EPPs and NEPs by families or classes. For instance, orange-yellow, brown-green, and purple-red spot coloration under UV light correspond to flavonoids, flavones, and anthocyanins, respectively.^35,58^ In addition, phenolic acids, such as *p*-coumaric acid, chlorogenic acid, ferulic acid, or caffeic acid, have been identified in several investigations as blue spots.^35,59^

According to reference colors, the qualitative identification of EPPs and NEPs from the different extracts of cherry pomace by phenolic families was carried out. HPTLC analysis showed that different classes of phenolic compounds were observed in the extracts collected by conventional extraction and each hydrolysis treatment detecting 29 compounds in total. Furthermore, as can be observed in Table S4 and Figure 1, the extracts collected by conventional extraction and alkaline and acid hydrolyses showed a higher number of spots (5, 8, and 6 compounds, respectively). This means that a wide range of phenolic compounds were retained in the residue of conventional extraction. Most of the separate compounds corresponded to phenolic acids and flavonoids or flavones by the blue and yellow-brown color of the bands (Figure 1). In this study, conventional extraction and alkaline hydrolysis allowed us to obtain a higher number of phenolic acids than the rest of the treatments, exhibiting a higher number of blue bands in the HPTLC separation process (see Figure 1). For instance, in the extracts recovered by conventional extraction, a phenolic acid with a blue spot (*R_f_* value of 0.17) with the highest intensity (283 AU) was observed (Figure S1). This phenolic acid could correspond to neochlorogenic or chlorogenic acid because these compounds have been found at high amounts in sweet cherry pomace.^60,61^ This compound, as well as the compound detected with a *R_f_* value of 0.70, was also detected in alkaline extract with higher intensity than conventional extract. In addition, TLC visualization showed that EAE extracts with the three enzymes employed presented the same spot with a *R_f_* value between 0.60 and 0.70 with intense blue color that was not present in the rest of the extracts. Promod HBN extract showed the lowest number of spots detected (2 compounds).

Concerning yellow spots, the alkaline extract showed different flavones in the separation by HPTLC with brown spots with *R_f_* values of 0.28, 0.49, and 0.60. In addition, the alkaline extract exhibited a yellow spot with the highest intensity (368 AU) with a *R_f_* value of 0.24, which could be epicatechin as this compound has been found as the flavonoid with the highest concentration in sweet cherry pomace.^60^ Acid hydrolysis allowed us to obtain a wide range of flavonoids because several brown and yellow color bands were exhibited in the HPTLC separation. On the other hand, the acid extract did not show phenolic acids. This may be due to the extreme pH values used in the extraction process and the fact that phenolic acids are unstable at these low pH values.

EAE extracts showed a lower number of bands than conventional, alkaline, and acid extracts. However, HPTLC visualization allowed determination of bands of different colors in EAE extracts. In fact, HPTLC combined with DART-HRMS presented a fast separation and tentative identification by families and specific EPPs and NEPs from cherry pomace.

### 3.3 Tentative Identification by DART-Orbitrap-HRMS of EPPs and NEPs Obtained by Conventional Extraction, Acid and Alkaline Hydrolyses, and EAE Using HBN Methodology

Table 2 summarizes the identification by DART-HRMS of EPPs obtained by conventional extraction from sweet cherry pomace as well as the identification of NEPs attained from the residue of conventional extraction by alkaline hydrolysis and EAE with Promod, Depol, and Pectinase enzymes under the optimal extraction conditions to produce HBN extracts. The acid extract could not be included in the analysis because ionization problems were observed probably due to interference from the acid solvent.

**Table 2:** Exact Mass Data and Intensity of Extractable and Nonextractable Polyphenols Identified by DART-Orbitrap-HRMS in Conventional, Alkaline, and Enzymatic (Promod, Depol, and Pectinase Enzymes) Extracts in Sweet Cherry Pomace

no.	compound	molecular formula	error (ppm)	measured mass [M – H]^−^	monoisotopic mass	conventional	alkaline	promod	depol	pectinase
**1**	dihydroxybenzoic acid	C_7_H_5_O_4_	4.08	153.0194	154.0266	53.76	4258.04		1329.21	3217.16
**2**	coumaric acid	C_9_H_7_O_3_	4.96	163.0390	164.0473	248.27	3199.32	1620.84		2458.34
**3**	vanillic acid	C_8_H_7_O_4_	6.15	167.0350	168.0422		15069.80		3104.49	5702.97
**4**	gallic acid	C_7_H_5_O_5_	4.89	169.0140	170.0215	413.03	31523.06	2907.67		5955.40
**5**	shikimic acid	C_7_H_9_O_5_	4.58	173.0452	174.0528				1297.64	
**6**	ferulaldehyde	C_10_H_9_O_3_	4.95	177.0553	178.0629		3986.94			
**7**	dihydroxycoumarin acid	C_9_H_5_O_4_	3.66	177.0188	178.0266	223.91			1262.51	
**8**	caffeic acid	C_9_H_7_O_4_	3.95	179.0346	180.0422	70.50	5912.06		1506.63	3268.08
**9**	syringaldehyde	C_9_H_9_O_4_	2.33	181.0500	182.0579	62.32	8533.31		3252.47	
**10**	methyl gallate	C_8_H_7_O_5_	5.55	183.0295	184.0371		14477.98	2381.43	1907.18	3488.29
**11**	quinic acid	C_7_H_11_O_6_	3.32	191.0554	192.0633		1433.59			
**12**	ferulic acid	C_10_H_9_O_4_	4.72	193.0502	194.0579	57.36	4484.96		2503.79	3730.41
**13**	syringic acid	C_9_H_9_O_5_	0.30	197.0449	198.0528		9707.51			3385.29
**14**	sinapaldehyde	C_11_H_11_O_4_	2.29	207.0657	208.0735	104.43	4282.13	1399.66		3067.83
**15**	hydroxyferulic acid	C_10_H_9_O_5_	1.67	209.0448	210.0528	61.62		1672.29		
**16**	pinocembrin	C_15_H_11_O_4_	–0.78	255.0649	256.0735	104.23		311.09		
**17**	vestitol	C_16_H_15_O_4_	–3.90	271.0954	272.1048				2702.58	2664.90
**18**	kaempferol/luteolin	C_15_H_9_O_6_	0.65	285.0391	286.0477	75.87	1115.14	382.52	1656.07	740.26
**19**	methyl naringenin	C_16_H_13_O_5_	–0.88	285.0755	286.0841			1308.70		
**20**	aromadendrin	C_15_H_11_O_6_	–0.29	287.0549	288.0633	58.66			3750.02	2021.65
**21**	(epi)catechin	C_15_H_13_O_6_	0.89	289.0701	290.0790	119.22	3089.82	1949.63	7063.75	3350.57
**22**	procyanidin B2	C_30_H_26_O_12_	0.26	289.0701	578.1424	582.88	3089.82	1949.63		3350.57
**23**	*p*-coumaroyl tartaric acid	C_13_H_11_O_8_	–0.37	295.0448	296.0532		1372.02	1352.99		2288.09
**24**	kaempferide	C_16_H_11_O_6_	–0.48	299.0538	300.0633		101370.00			
**25**	quercetin	C_15_H_9_O_7_	–0.84	301.0340	302.0426	149.16	955.28	697.94	2055.55	857.03
**26**	taxifolin	C_15_H_11_O_7_	–0.11	303.0499	304.0583	208.81	2354.51	1622.33	3999.58	2064.73
**27**	(epi)gallocatechin	C_15_H_13_O_7_	–3.29	305.0652	306.0739	1067.65	3429.07	2353.77		
**28**	caftaric acid	C_13_H_11_O_9_	–2.63	311.0395	312.0481			913.47		
**29**	dihydromyricetin	C_15_H_11_O_8_	–1.96	319.0442	320.0532				3206.39	
**30**	myricetin	C_15_H_9_O_8_	–0.06	317.0287	318.0375	107.27	1111.02	639.31		857.97
**31**	vanillic acid-hexoside	C_18_H_33_O_5_	–4.02	329.2318	330.0950		940.17			
**32**	glucogallic acid	C_13_H_15_O_10_	–0.72	331.0656	332.0743		1299.69			
**33**	methoxytaxifolin	C_16_H_13_O_8_	–2.68	333.0596	334.0688	113.61			3471.34	
**34**	coumaroylquinic acid	C_16_H_17_O_8_	–0.35	337.0915	338.1001		2197.38			
**35**	chlorogenic acid	C_16_H_19_O_9_	0.94	354.0950	355.1029		1387.13			
**36**	retusin	C_19_H_17_O_7_	0.38	357.0972	358.1052	364.02				824.83
**37**	glucosyringic acid	C_15_H_19_O_10_	–1.06	359.0969	360.1056	286.29				367.36
**38**	feruloylquinic acid	C_17_H_19_O_9_	0.82	367.1018	368.1107		1499.35			
**39**	sinapoylglucose	C_17_H_21_O_10_	–0.18	385.1119	386.1212		887.76			

**Figure 2:**
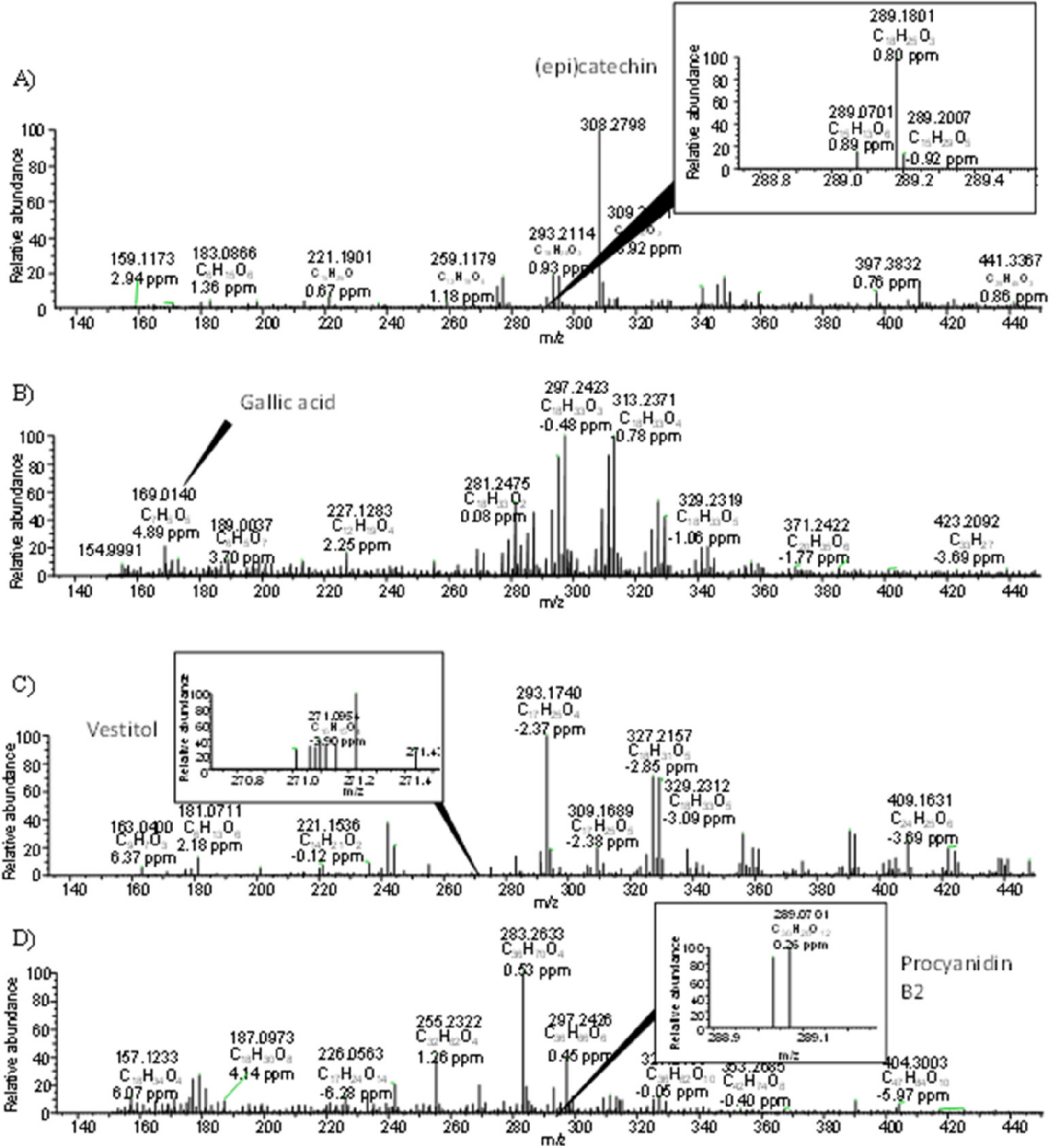
Mass spectrum ([M – H]^−^) of NEPs from sweet cherry pomace of (A) (epi)catechin from EAE with Depol enzyme extract, (B) gallic acid from alkaline extract, (C) vestitol from EAE with Depol enzyme extract, and (D) procyanidin B2 from EAE with Pectinase enzyme extract.

As can be seen in Table 2, a total of 39 phenolic compounds were tentatively identified by DART-Orbitrap-HRMS in sweet cherry pomace extracts. The highest number of NEPs detected corresponded to the alkaline extract where a total of 27 NEPs were identified. Four phenolic compounds were found in common in all extracts: kaempferol/luteolin (number 18), (epi)catechin (number 21), quercetin (number 25), and taxifolin (number 26). In particular, (epi)catechin with a molecular ion at *m*/*z* 289.0701 [M – H]^−^ presented the highest intensity in the extracts performed by EAE with Depol enzyme (Figure 2A). Several researchers observed that catechin and epicatechin are present in sweet cherries at high concentrations. Generally, epicatechin is more concentrated in sweet cherries than catechin.^60,63,64^ These compounds were detected in sweet cherry pulp as well as in its byproducts such as stems.^65^ Moreover, (epi)gallocatechin (number 27) with a molecular ion at *m*/*z* 305.0652 [M – H]^−^ was the most intense phenolic compound identified in the conventional extract. Nevertheless, this compound was observed with higher intensity in the alkaline extract than in the conventional extract. (Epi)gallocatechin has also been identified in sweet cherry pulp and stems.^66,67^

A total of 20 phenolic acids were identified in DART-Orbitrap-HRMS analysis of sweet cherry pomace extracts, being the majority class of phenolic compounds determined in the analysis. Among them, gallic acid (number 4) with a molecular ion at *m*/*z* 169.0140 [M – H]^−^ was tentatively identified as the most intense phenolic acid observed in the extracts (Figure 2B). In particular, this compound was observed in alkaline and EAE with Promod enzyme extracts at a high intensity (see Table 2). Additionally, gallic acid was detected with the highest intensity in the extracts collected by EAE with the Pectinase enzyme. The presence of gallic acid has been described in sweet cherry pulp as well as in stems, although it depends on the variety of sweet cherries studied.^60,66,68^

Dihydroxybenzoic acid (number 1) with a molecular ion at *m*/*z* 153.0194 [M – H]^−^ was tentatively identified in all extracts. Commonly, different hydroxybenzoic acids, such as hydroxybenzoic acid derivative, protocatechuic acid aglycone, 2,5-dihydroxybenzoic acid, and *p*-hydroxybenzoic acid, were detected in sweet cherry pulp and stems.^67,69,70^

On the other hand, an isoflavone with a molecular ion at *m*/*z* 271.0954 [M – H]^−^ was tentatively identified as vestitol (number 17) in EAE extracts with Depol and Pectinase enzymes (see Table 2, Figure 2C). Isoflavones are commonly found in legumes. However, these compounds have also been found in different fruit peels such as *Mangifera pajang* Korterman peels or different varieties of passion fruits such as *Passiflora edulis*, *Passiflora ligularis*, and *Passiflora mollissima* peels.^71,72^

Concerning procyanidins, the precursor of procyanidin B2 (number 22, Figure 2D) with a molecular ion at *m*/*z* 289.0701 [M – H]^−^ with charge 2 in all extracts, excepting EAE with Depol enzyme extract, was tentatively identified as the NEP with the highest molecular weight. This compound has been detected in the pulps of different varieties of sweet cherries.^60,64^

To our knowledge, this is the first time that NEPs from sweet cherry pomace were separated and identified by families by HPTLC-UV/vis and directly identified by DART-Orbitrap-HRMS. In addition, to check the extraction efficiency of the different treatments employed in this work to release EPPs and NEPs from sweet cherry pomace, the residues from conventional extraction and hydrolysis treatments were analyzed by DART-Orbitrap-HRMS. As can be seen in Table S5, hydrolysis treatments were efficient in the release of NEPs from the residue of conventional extraction since the signal intensities of NEPs identified by DART-HRMS in the residues of hydrolysis treatments were lower than in the extracts.

### 3.4 Evaluation of Biological Activities of Extractable Phenolic Compounds and NEPs from Sweet Cherry Pomace

#### 3.4.1 Antihypertensive Capacity

Table 3 shows the concentration of the extracts necessary for 50% inhibition of ACE activity (expressed as IC_50_) the extracts with the highest antihypertensive capacity being the ones with the lowest IC_50_ values. In general, EAE extracts showed higher antihypertensive capacity than those from conventional extraction and acid and alkaline hydrolysis. Depol HBN extract presented the highest antihypertensive capacity but did not show statistical differences with Pectinase TPA extract. By contrast, Promod HBN extract showed the lowest antihypertensive capacity compared with the rest of EAE extracts with the three enzymes studied coinciding with conventional extraction and alkaline hydrolysis.

**Table 3:** Antioxidant Capacity (DPPH (EC_50_, (μg/mL)/Sample), TEAC (μmol Trolox/g Sample), and Inhibition of Hydroxyl Radical Assays (%)) and Antihypertensive Capacity (ACE Inhibition Assay (IC_50_, g of Extraction Residue/mL)) Obtained by Different Extraction Methods from Cherry Pomace⊗

sample	DPPH	TEAC	^•^OH	IC_50_
conventional	527 ± 1^c^	3.27 ± 0.01^h^	8.9 ± 0.1^j^	0.15 ± 0.01^f^
acid	1523 ± 28^h^	9 ± 1^d,e^	5.4 ± 0.04^k^	0.220 ± 0.004^g^
alkaline	770 ± 25^g^	6.4 ± 0.1^f,g^	11.5 ± 0.7^i^	0.16 ± 0.02^f^
Pectinase HBN	713 ± 10^f^	5.69 ± 0.07^f,g,h^	75.6 ± 0.2^a^	0.07 ± 0.02^d^
Pectinase TPA	635 ± 31^e^	3.83 ± 0.02^g,h^	63.1 ± 0.5^b^	0.011 ± 0.004^a,b^
Pectinase HB	440 ± 13^a^	5.48 ± 0.03^f,g,h^	52.5 ± 1.1^e^	0.024 ± 0.003^b,c^
Promod HBN	588 ± 24^d^	11.09 ± 0.04^c,d^	54.4 ± 0.2^d^	0.161 ± 0.003^f^
Promod TPA	736 ± 34^e^	19 ± 1^b^	58.21 ± 0.04^c^	0.149 ± 0.004^e,f^
Promod HB	599 ± 20^a^	38 ± 5^a^	58.6 ± 1.5^c^	0.130 ± 0.003^e^
Depol HBN	608 ± 6^d,e^	10.4 ± 0.3^c,d^	43.8 ± 0.3^f^	0.00080 ± 0.00007^a^
Depol TPA	480 ± 9^b^	7.00 ± 0.05^e,f^	33.9 ± 0.1^h^	0.03 ± 0.01^c^
Depol HB	407 ± 2^a^	12.0 ± 0.3^c^	36.2 ± 0.3^g^	0.023 ± 0.008^b,c^

⊗ Letters (a, b, c, d, e, f, g, h, i, j, k) show the significant differences among extraction methods of NEPs (*p* ≤ 0.05).

#### 3.4.2 Antioxidant Capacity

The results obtained from DPPH, TEAC, and hydroxyl radical assays are summarized in Table 3, showing statistical differences (*p* ≤ 0.05) among extraction methods in all assays. HB extracts showed the highest antioxidant values compared with HBN and TPA extracts with each enzyme employed in DPPH and TEAC assays. EAE extracts showed higher antioxidant capacity than alkaline and acid hydrolysis highlighting Promod HB and Depol HB extracts in DPPH assay. Also, the highest antioxidant capacity was obtained from Promod HB extract in the TEAC assay. However, DPPH and TEAC are spectrophotometric assays where the radicals employed are not generated in our bodies. For this reason, the results are limited because they do not reproduce the physiological situation. In this sense, hydroxyl radical is a potent reactive oxygen species in the biological system. The estimation of the inhibition of hydroxyl radical could provide an approximation of the antioxidant effect of the extracts in our body. Results in Table 3 show that the inhibition of hydroxyl radical depends on the hydrolysis treatment and extraction conditions employed to obtain NEPs (*p* ≤ 0.05). This method demonstrated high antioxidant capacity in cherry pomace extracts obtained by EAE. Pectinase HBN extract showed the highest hydroxyl radical inhibition. By contrast, hydrolysis acid extract showed the lowest hydroxyl radical inhibition.

To verify the *in vivo* biological activity of NEPs, a *C. elegans* model was used for the first time to evaluate the antioxidant capacity of the extracts on this nematode. The antioxidant power was evaluated by inducing oxidative stress in *C. elegans* with H_2_O_2_ due to the effect of this pro-oxidant on the lifespan and mortality of the worm. This way, the antioxidant effect of EPPs and NEPs obtained by conventional, acid, alkaline, and EAE was proportional to the survival rate of the worms.

**Figure 3:**
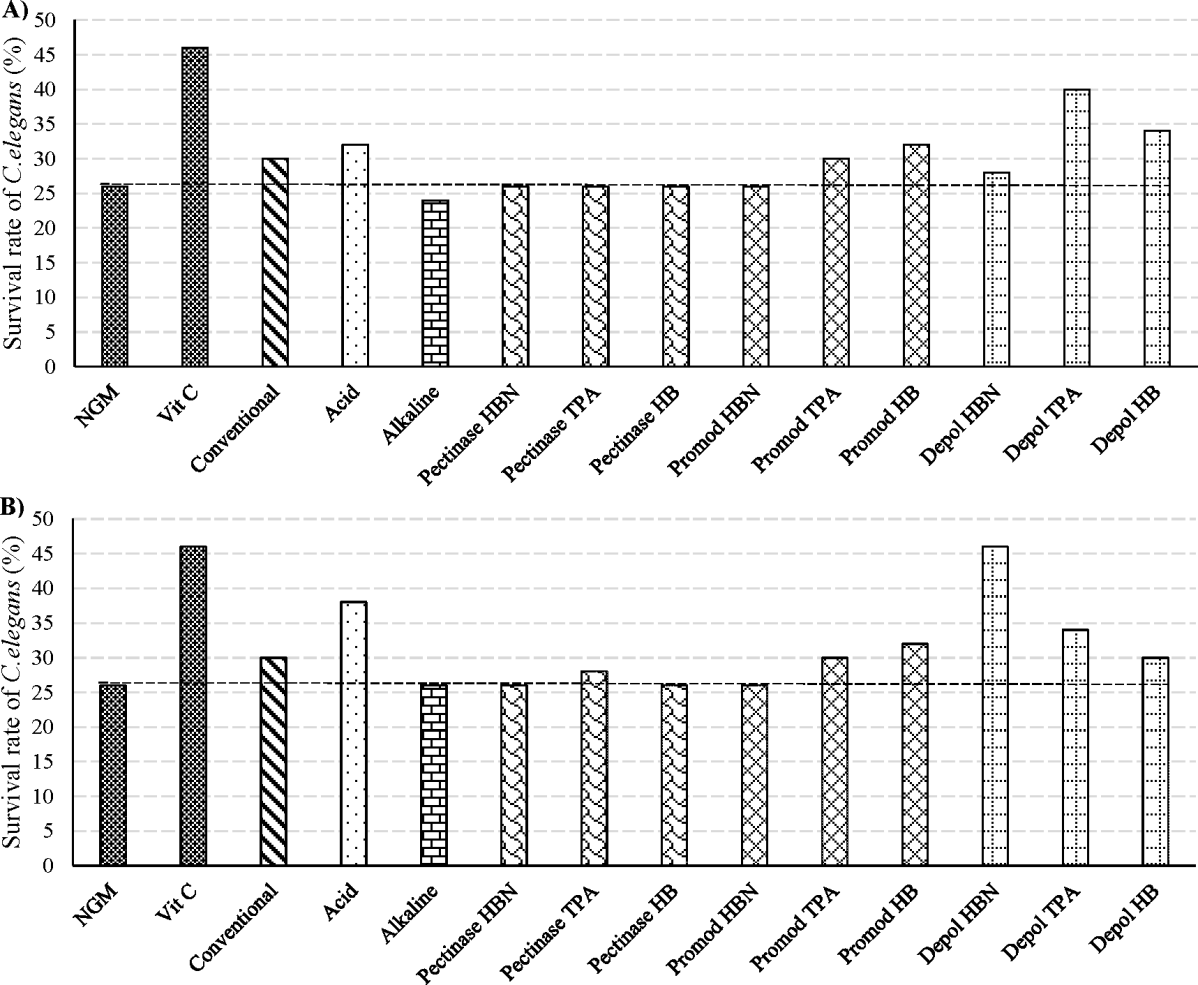
Effects of cherry pomace nonextractable polyphenol hydrolysates on the survival rate of *C. elegans* in response to H_2_O_2_-induced oxidative stress expressed as the % survival rate of *C. elegans* in normal medium (NGM), medium containing 10 μg/mL vitamin C, or different nonextractable polyphenol hydrolysates from cherry pomace at (a) 100 μg/mL extract and (b) 400 μg/mL extract.

Figure 3A,B shows the survival percentage of the nematode population under oxidative stress conditions when 100 μg/mL and 400 μg/mL, respectively of different sweet cherry pomace extracts were added. As it can be seen in Figure 3A, Depol TPA extract was the most antioxidant extract as the survival rate of *C. elegans* increased by 14% compared to control (NGM), followed by Depol HB extract (12% survival rate). By contrast, acid hydrolysis and EAE with Promod enzyme to obtain HB extracts showed an increase in the survival of *C. elegans* by 6%, as did the extracts obtained by conventional extraction and Promod TPA. No protective activity against stress could be determined with the rest of the extracts. On the other hand, when a higher extract concentration (400 μg/mL) was used, Depol HBN extract presented the highest antioxidant capacity, increasing the survival rate of *C. elegans* by 20% compared to the control (NGM) (Figure 3B). This increase in the survival rate of *C. elegans* was comparable to the one observed with the positive control (vitamin C, 10 μg/mL). Moreover, acid hydrolysis and Depol TPA extracts displayed high antioxidant activity. The extracts collected by EAE with Depol and by acid hydrolysis showed higher antioxidant activity at both concentrations, 100 and 400 μg/mL, increasing the survival rate of *C. elegans* by around 6–20% compared with the NGM control.

The high antioxidant capacity of Depol HBN and TPA extracts evaluated in *C. elegans* showed a correlation with the TPC measured by FC assay, Depol HBN and TPA extracts showing the highest TPC content. These results suggest that during EAE with Depol enzyme, phenolic compounds different from proanthocyanidins, which are not detected in PA assays, are released from the extraction residue and showed an important *in vivo* antioxidant capacity.

### 3.5 Antiaging Capacity

In order to measure the antiaging capacity of the extracts from cherry pomace, two methods were employed. The elastase inhibition activity method was used to evaluate the *in vitro* antiaging capacity of the extracts to be compared with the results obtained by the *in vivo* health span method.

The elastase activity exhibited by EPPs and NEPs is shown in Table 4. The acid extract showed the highest inhibition of elastase activity, but the value was lower than that with a concentration of 0.7 mg/mL of epicatechin. Regarding EAE, Promod enzyme resulted in the HBN extract with the highest inhibition of elastase activity, higher than that from conventional extraction.

**Table 4:** Antiaging Capacity (Elastase Inhibition Activity Assay (%)) and Neuroprotective Capacity (Anticholinesterase Inhibitory Activity (%)) Obtained by Different Extraction Methods from Cherry Pomace⊗

sample	elastase inhibition	AChE inhibition
conventional	52 ± 1^d^	28.1 ± 0.3^h^
acid	80.8 ± 0.9^a^	76 ± 4^a^
alkaline	69.5 ± 0.6^b^	35.5 ± 0.8^f^
Pectinase HBN	33.3 ± 0.5^g^	55.1 ± 0.4^c^
Pectinase TPA	14 ± 1^j^	55.1 ± 0.3^c^
Pectinase HB	50.2 ± 0.5^d^	69.6 ± 0.8^b^
Promod HBN	57.7 ± 0.5^c^	32 ± 2^g^
Promod TPA	34 ± 1^g^	54.9 ± 0.5^c^
Promod HB	41 ± 2^e^	51 ± 2^d^
Depol HBN	23 ± 1^i^	36 ± 3^f^
Depol TPA	38 ± 1^f^	56.1 ± 0.4^c^
Depol HB	31 ± 1^h^	44.0 ± 0.7^e^

⊗ Letters (a, b, c, d, e, f, g, h, i, j) show the significant differences among extraction methods of NEPs (*p* ≤ 0.05).

**Figure 4:**
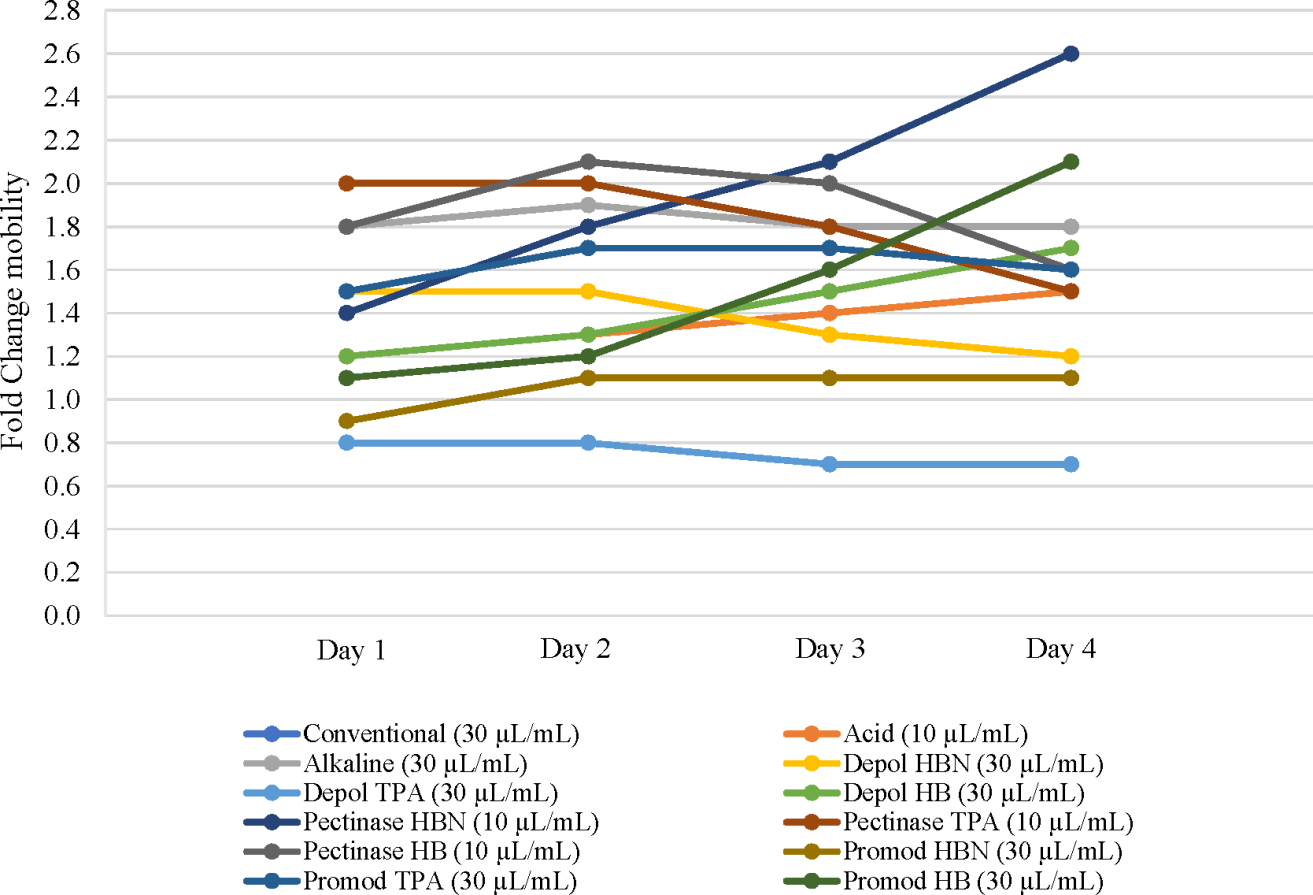
Effect of nonextractable polyphenol hydrolysates on *C. elegans* health span. Fold Change mobility values (activity treatment/activity control) are represented for the different feed conditions.

On the other hand, the *in vivo* evaluation of the antiaging capacity of NEPs was evaluated for the first time by the mobility of *C. elegans* under the different extracts as an aging-related parameter. The activity of nematodes treated with different extracts was measured daily and compared with nematodes in control feed conditions (NGM) during the first 4 days of adulthood (Table S6). All extracts from sweet cherry pomace showed a positive effect on mobility using 30 μL/mL of extract except acid and Pectinase extracts, which exhibited a higher effect employing 10 μL/mL of extract (see Table S6). Therefore, the extracts were evaluated at the optimal concentration observed in the screening test. As can be observed in Figure 4, except for Depol TPA extract, all extracts provided a fold change value >1, indicating a positive effect on mobility. Moreover, among extracts tested, Promod HBN showed the lowest effect, with fold change values near to 1 and below the activity of nematodes treated with the conventional extract. Interestingly, nematodes treated with Pectinase HB, Pectinase TPA, alkaline, and Pectinase HBN extracts caused an increase in worm’s mobility (fold change values 1.8–2.6).

*In vitro* and *in vivo* assays showed different results on the antiaging capacity because these assays analyzed different antiaging parameters. The *in vitro* assay was focused on the determination of the elastase inhibition capacity to prevent the drastic decrease in skin elasticity with age, while *in vivo* assay determines the prevention of the reduction in the mobility caused by the aging process. However, both assays showed that NEPs from sweet cherry pomace provide higher antiaging capacity than EPPs recovered by conventional extraction. Promod HBN extract was distinguished by high antiaging capacity *in vitro* and *in vivo*.

### 3.6 Neuroprotective Capacity

The AChE inhibitory activity of the extracts was evaluated *in vitro* by Ellman’s method to determine the potential of the extracts to revert the cholinergic deficit in Alzheimer’s disease. As can be seen in Table 4, the acid hydrolysis extract showed the highest AChE inhibition with a higher neuroprotective capacity than galantamine at a concentration of 100 μM. EAE extracts showed higher AChE inhibition than the alkaline extract, except EAE with Promod enzyme to obtain HBN extracts, which showed inhibition of 32% ± 2%. By contrast, the conventional extract showed the lowest acetylcholinesterase inhibition.

**Figure 5:**
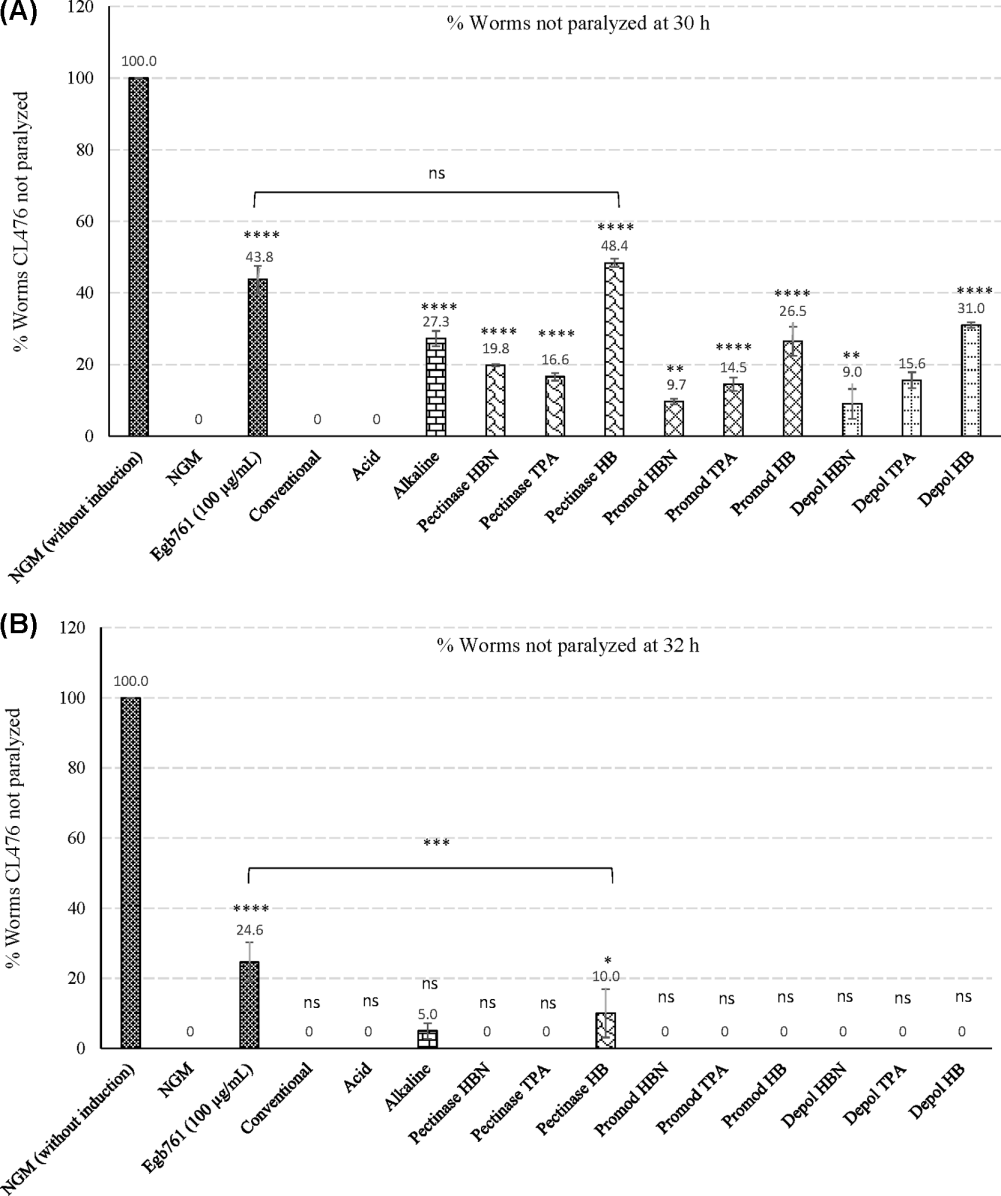
Percentage of not paralyzed CL4176 scored at (A) 30 h and (B) 32 h treated with 12 different nonextractable polyphenol hydrolysates at a dose of 300 μL (50 μL for acid extract). ****p ≤ 0.0001, **p ≤ 0.01; ns, not significant. Statistical comparison vs NGM condition.

The neuroprotective capacity of different NEPs extracts was evaluated for the first time *in vivo* in *C. elegans* by a model inducing paralysis of nematodes by upshifting temperature, which induces the expression of the human amyloid β-peptide. Figure S2 shows the percentage of CL4176 worms not paralyzed for 24, 26, 28, 30, and 32 h at three different doses of extracts (100, 200, and 300 μL) in NGM medium compared with a positive control with EGb 761, NGM with the induction of paralysis without extracts, and NGM without induction. Acid extract was added at lower doses (10, 25, and 50 μL) since this extract at higher volumes affected the egg-laying and eggs were not able to hatch (Figure S2B). Results showed that Pectinase HB, alkaline, and Depol HB extracts exhibited the most neuroprotective effect: the percentage of nonparalyzed worms at 28 and 30 h in groups treated at a dose of 300 μL was higher than that with the rest of the extracts (Figures S2 and 5A). The protective effect of Pectinase HB extracts was higher than the positive control at 30 h but without significant differences (see Figure 5A). This extract also showed a protective effect at 32 h, but it was lower than that with the positive control (Figure 5B). By contrast, the acid extract did not show any protective effect.

Even though acid extract provided a positive *in vitro* neuroprotective effect, a detrimental effect was observed *in vivo* on *C. elegans* (and possibly in our body) due to the low pH of the extract. Thus, NEPs released from EAE with Pectinase to obtain HB extracts exhibited the most neuroprotective effect both *in vitro* and *in vivo*.

### 3.7 Cytotoxic and Proliferative Effects in HepG2, HFF-1, SKOV3, and HT-29 Cell Lines of EPPs Obtained by Conventional Extraction and NEPs Obtained by Alkaline Hydrolysis and EAE from Sweet Cherry Pomace

Some natural compounds may also cause health problems in the human body due to their proliferative and cytotoxic effects.^37^ Therefore, safety is critical in the development of novel products for the pharmaceutical, cosmetic, or food industries.^73^ One of the most metabolically competent cell lines for cytotoxic assays is the human hepatocarcinoma HepG2 because the majority of toxicological studies indicate that toxic effects derived from natural compounds are associated with hepatotoxicity.^73,74^ In this manner, the HepG2 cell line provides the closest *in vitro* model to the human liver in cytotoxic assays.^73^ Thus, it is advisable to evaluate the toxicity of natural compounds by using different cell lines. The cytotoxicity of conventional, alkaline, acid, and EAE extracts was evaluated. In particular, HBN extracts attained by EAE were selected as the most representative extracts to evaluate the cytotoxic effect of EAE extracts. The first preliminary study allowed observing that the acid extract presented a very high cytotoxic effect hindering the measure of its absorbance.

**Figure 6:**
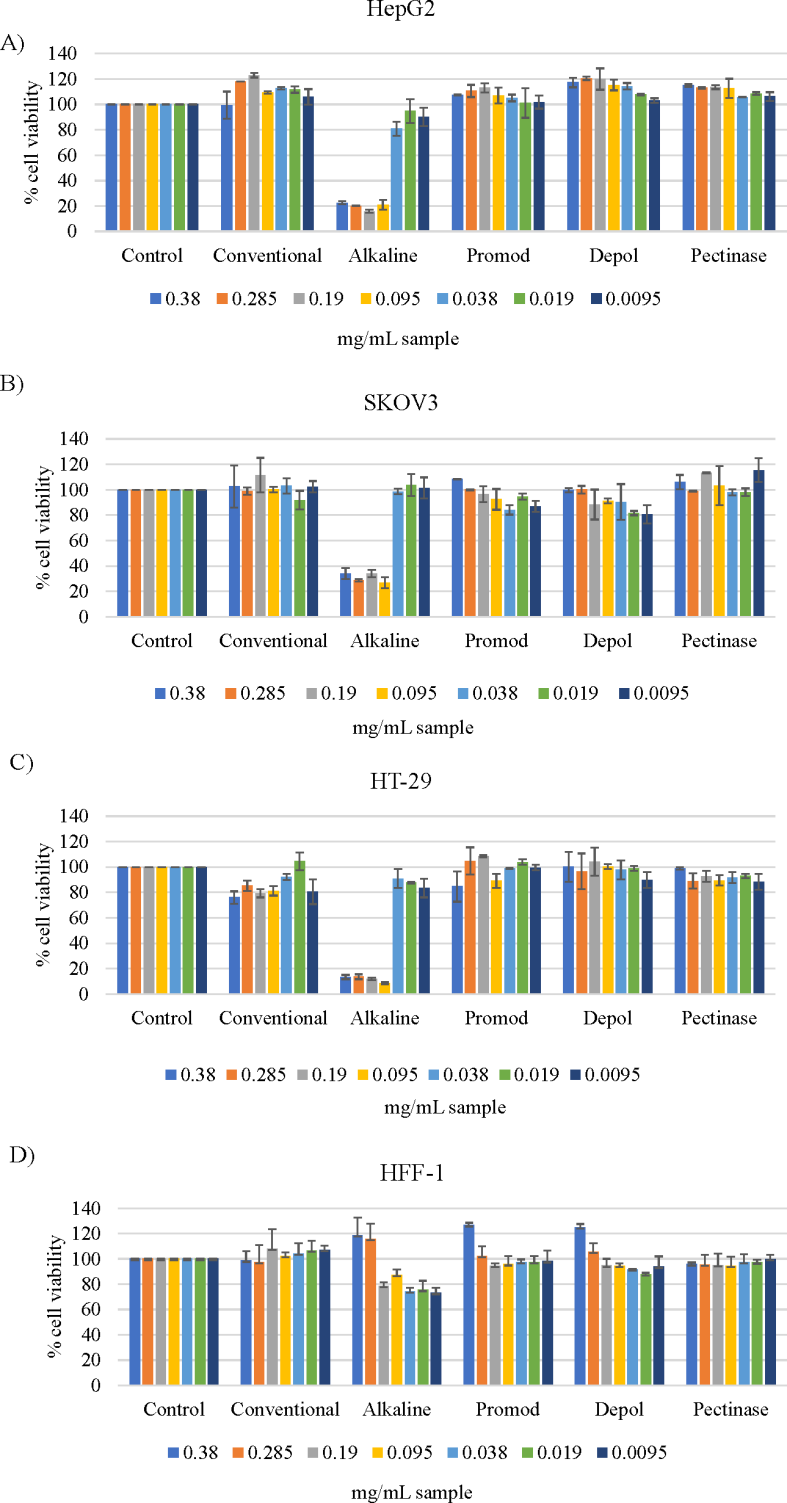
Cytotoxic and proliferative effects of nonextractable polyphenol hydrolysates obtained by conventional extraction, alkaline hydrolysis, and EAE with Promod, Depol, and Pectinase enzymes from sweet cherry pomace at different concentrations (0.3800–0.0095 mg/mL) on (A) hepatocarcinoma HepG2, (B) human ovarian cancer SKOV3, (C) colon adenocarcinoma HT-29, and (D) primary dermal fibroblast HFF-1 cell lines.

Figure 6 shows the cytotoxic effect on HepG2, SKOV3, and HT-29 cancer cell lines, as well as HFF-1 primary cell line, of different concentrations (0.3800–0.0095 mg/mL sample) of conventional, alkaline, and EAE extracts from sweet cherry pomace. As can be seen in Figure 6A,B,C, a cytotoxic effect was observed with alkaline extract on HepG2, SKOV3, and HT-29 cell lines, while the highest concentration of the alkaline sample (>0.2850 mg/mL) did not present a cytotoxic effect on the HFF-1 primary cell line (see Figure 6D). However, this extract also exhibited a proliferative effect on the HFF-1 cell line as concentrations of 0.3800 and 0.2850 mg/mL sample increased the cell viability compared with control. These results suggested that cell lines differed in their sensitivity to the same samples, which may depend on multiple cell type-specific signaling cascades of each cell line as well as their transcription factor activities. The proliferative effect observed with the alkaline sample treatment could be due to the presence of determined phytochemicals released during alkaline hydrolysis treatment, which cause injury to the liver, colon, ovary, and skin.

On the other hand, a concentration of 0.2850 mg/mL of all samples did not show cytotoxic and proliferative effects on SKOV3, except for alkaline extracts (see Figure 6B). Regarding the HT-29 cell line, the highest and the lowest concentrations of extracts attained by EAE with Pectinase and EAE with Promod did not show cytotoxic effects (see Figure 6C).

The low cytotoxicity and nonproliferative effect of the extracts achieved by EAE with Promod enzyme in all cell lines studied (HepG2, HFF-1, SKOV3, and HT-29) suggested that this extract could be the most suitable to be included as a bioactive ingredient in future formulations for the elaboration of products with beneficial health properties.

## 4 Conclusions

This work presents for the first time an *in vivo* evaluation of the antioxidant, antiaging, and neuroprotective capacities of NEP extracts from cherry pomace using *C. elegans* as an experimental animal model where EAE extracts presented the highest biological activities. Depol HBN, Promod HB, Pectinase HBN, and Depol TPA extracts were highlighted as the most bioactive extracts *in vitro* and *in vivo*. Nevertheless, EAE with Promod enzyme extract was the only one that did not present a cytotoxic effect on HepG2, HFF-1, SKOV3, and HT-29 cell lines. The fast HPTLC analytical method to separate extractable polyphenols and NEPs allowed identification of these compounds by families. Furthermore, the rapid and tentative identification of up to 39 NEPs in sweet cherry pomace was carried out for the first time by DART-Orbitrap-HRMS. To our knowledge, some phenolic compounds such as vestitol, scopoletin, or procyanidin B2 had not previously been identified in this fruit byproduct. *In vitro* and *in vivo* experiments as well as HPTLC and DART-Orbitrap-HRMS analysis revealed that conventional extraction is an inefficient technique to extract phenolic compounds since phenolic compounds with important biological properties were retained in the extraction residue. EAE is a promising alternative to release NEPs, providing extracts with high biological capacities. In fact, it permitted us to obtain nontoxic extracts with high *in vivo* antioxidant, antiaging, and neuroprotective capacities.

## Supporting Information Available

Optimal conditions to obtain HBN, TPA and HB extracts by EAE, theoretical values of TPC (Folin–Ciocalteu method) and total PA content and antioxidant and antihypertensive capacity (ACE inhibition method) obtained under optimal EAE conditions, *R_f_* values, peak areas, and wavelengths at absorption maximum of separated phenolic compounds by HPTLC, exact mass data and intensity of nonextractable polyphenols, fold change values of the mobility of *C. elegans* treated with the different extracts at three doses, effect of extracts on body paralysis delay, and UV absorption chromatograms at 280 nm with *R_f_* values of extracts (PDF)
