# Supplementary material for: Composition of Nonextractable Polyphenols from Sweet Cherry Pomace Determined by DART-Orbitrap-HRMS and Their In Vitro and In Vivo Potential Antioxidant, Antiaging, and Neuroprotective Activities
Source: J Agric Food Chem. 2022 Jun 22;70(26):7993–8009. doi: 10.1021/acs.jafc.2c03346 (PMC9264388; doi:10.1021/acs.jafc.2c03346)

SUPPLEMENTARY MATERIAL

COMPOSITION OF NON-EXTRACTABLE POLYPHENOLS FROM SWEET CHERRY POMACE BY DART-ORBITRAP-HRMS AND THEIR *IN VITRO* AND *IN VIVO* POTENTIAL ANTIOXIDANT, ANTIAGING, AND NEUROPROTECTIVE ACTIVITIES

Gloria Domínguez-Rodríguez[1,2], Daniel Ramón[3], Patricia Martorell[3], Merichel Plaza[1,4], María Luisa Marina[1,4*]

[1]Universidad de Alcalá, Departamento de Química Analítica, Química Física e Ingeniería Química, Facultad de Ciencias, Ctra. Madrid-Barcelona Km. 33.600, 28871 Alcalá de Henares (Madrid), Spain.

[2]Mendel University in Brno, Department of Chemistry and Biochemistry, Zemedelska 1, CZ-613 00 Brno, Czech Republic

[3]Archer Daniels Midland, Nutrition, Health&Wellness, Biopolis S.L. Parc Scientific Universitat de València, C/Catedrático Agustín Escardino Benlloch, 9, Paterna, 46980 Valencia, Spain

[4]Universidad de Alcalá, Instituto de Investigación Química Andrés M. del Río (IQAR), Ctra. Madrid-Barcelona. Km. 33.600, 28871 Alcalá de Henares (Madrid), Spain.

[*]Corresponding author: María Luisa Marina

Email: mluisa.marina@uah.es

Tel: (+34) 918854935

Table S1. Optimal conditions (enzyme concentration, extraction time, temperature, and pH) to obtain HBN, TPA and HB extracts from the extraction residue of sweet cherry pomace by EAE with Depol, Promod, and Pectinase enzymes.

	Optimal extraction conditions			
**HBN extracts**	**Enz. Conc. (µL/g)**	**Time (min)**	**Temp. (°C)**	**pH**
Promod enzyme	90	40	70	10.0
Depol enzyme	140	40	70	10.0
Pectinase enzyme	2	18.4	70	10.0
**TPA extracts**				
Promod enzyme	137.4	5.8	80	10.0
Depol enzyme	90	40	76	10.0
Pectinase enzyme	8.3	27.4	66	10.0
**HB extracts**				
Promod enzyme	138.8	40	60	10.0
Depol enzyme	40	5	65	10.0
Pectinase enzyme	0.5	5	68	9.0

Table S2. Theoretical values of TPC (Folin-Ciocalteu method) and total PA content (DMAC, vanillin, and butanol/HCl assays) obtained under the optimal EAE conditions.

Theoretical values	Promod enzyme TPA			Depol enzyme TPA			Pectinase enzyme TPA		
	**Optimum value**	**Lower**	**Upper**	**Optimum value**	**Lower**	**Upper**	**Optimum value**	**Lower**	**Upper**
**Folin-Ciocalteau method** (mg GAE/100 g sample)	188.7	157.4	220	39.7	31.2	48.3	115.7	99.8	131.6
**DMAC assay** (mg epicatechin/100 g sample)	0.2	-0.002	0.4	0.4	0.4	0.5	0.1	0.02	0.2
**Vanillin assay** (mg epicatechin/100 g sample)	65.2	38.5	91.9	40.9	24	57.8	24.6	21.1	28.2
**Butanol/HCl assay** (mg epicatechin/100 g sample)	43.1	18.4	100.7	27.6	20.5	34.6	27.7	21.6	33.7

Table S3. Theoretical values of antioxidant capacity (DPPH, TEAC, and capacity to inhibit the formation of hydroxyl radical methods) and antihypertensive capacity (ACE inhibition method) obtained under optimal EAE conditions.

Theoretical values	Promod enzyme HB			Depol enzyme HB			Pectinase enzyme HB		
	Optimum value	Lower	Upper	Optimum value	Lower	Upper	Optimum value	Lower	Upper
**DPPH method** (EC_{50}, µg/mL sample)	1061.9	539.8	1583.9	594.3	109	1079.6	533.3	432.2	634.5
**TEAC method** (µmol Trolox/g sample)	99.9	37.7	162.2	26.1	8.9	43.5	91.8	-22.3	205.9
**Capacity to inhibit formation hydroxyl radical** (% inhibition)	106.6	89.7	123.5	106.1	84	128.2	26.7	17	36.5
**Antihypertensive capacity** (IC_{5O} g of extraction residue/mL)	0.25	0.16	0.33	0.13	0.079	0.18	0.23	0.16	0.29

Table S4. *Rf* values, peak areas in absorbance units [AU], and wavelengths at absorption maximum (nm) of separated phenolic compounds by HPTLC from conventional extraction, acid, alkaline, and EAE extracts from sweet cherry pomace.

Extraction	Nº of spots found	*Rf*	Absorbance (nm)	Peak area [AU]
Conventional extraction	5	0.08	200	15
		0.17	203	283
		0.73	200	82
		0.80	200	82
		0.89	252	95
Alkaline hydrolysis	8	0.17	204	570
		0.24	311	368
		0.28	309	148
		0.49	200	86
		0.60	200	77
		0.70	270	113
		0.87	200	123
		0.95	257	162
Acid hydrolysis	6	0.16	290	693
		0.31	200	94
		0.44	292	126
		0.69	286	400
		0.76	289	142
		0.86	285	158
Enzymatic hydrolysis by Depol	4	0.17	200	197
		0.20	267	100
		0.90	267	100
		1.00	200	113

Enzymatic hydrolysis by Promod	2	0.16	200	95
		0.91	200	430
Enzymatic hydrolysis by Pectinase	4	0.17	200	98
		0.55	201	444
		0.96	200	52

Table S5. Exact mass data and intensity of non-extractable polyphenols identified by DART-Orbitrap-HRMS in residues of conventional, alkaline, and enzymatic extracts (obtained with Promod, Depol, and Pectinase enzymes) in sweet cherry pomace (C: conventional extraction, A: alkaline hydrolysis, P: EAE with Promod enzyme, D: EAE with Depol enzyme, Pe: EAE with Pectinase enzyme).

N°	Compound	Molecular formula	Error (ppm)	Measured mass [M-H]$^-$	Monoisotopic mass	C-Residue	A-Residue	P-Residue	D-Residue	Pe-Residue
**1**	Dihydroxybenzoic acid	$C_7H_5O_4$	4.08	153.0194	154.0266	79.46	128.31	28180.13	120.94	107.47
**2**	Coumaric acid	$C_9H_7O_3$	4.96	163.0390	164.0473	100.31	56.21	2946.08	60.59	35.49
**3**	Vanillic acid	$C_8H_7O_4$	6.15	167.0350	168.0422	72.06	51.17	708.62	96.84	121.24
**4**	Gallic acid	$C_7H_5O_5$	4.89	169.0140	170.0215				38.66	85.7
**5**	Shikimic acid	$C_7H_9O_5$	4.58	173.0452	174.0528			1115.73		
**6**	Caffeic acid	$C_9H_7O_4$	3.95	179.0346	180.0422	176.15				
**7**	Methyl gallate	$C_8H_7O_5$	5.55	183.0295	184.0371	67.54			88.58	257.57
**8**	Quinic acid	$C_7H_{11}O_6$	3.32	191.0554	192.0633	200.13	442.92	14775.92	150.86	526.06
**9**	Ferulic acid	$C_{10}H_9O_4$	4.72	193.0502	194.0579	58.79	37.07	6950.41	73.97	128.98
**10**	Syringic acid	$C_9H_9O_5$	0.30	197.0449	198.0528	76.81	108.33	20028.67	111.67	127.48
**11**	Sinapaldehyde	$C_{11}H_{11}O_4$	2.29	207.0657	208.0735			4120.45		44.48
**12**	Hydroxyferulic acid	$C_{10}H_9O_5$	1.67	209.0448	210.0528	70.42	149.35	27093.66	287.56	142.62
**13**	Pinocembrin	$C_{15}H_{11}O_4$	-0.78	255.0649	256.0735			2462.15		
**14**	Vestitol	$C_{16}H_{15}O_4$	-3.90	271.0954	272.1048	36.12				
**15**	Kaempferol/luteolin	$C_{15}H_9O_6$	0.65	285.0391	286.0477	38.32				
**16**	Aromadendrin	$C_{15}H_{11}O_6$	-0.29	287.0549	288.0633	115.13		2926.98	42.45	122.66
**17**	(Epi)Catechin	$C_{15}H_{13}O_6$	0.89	289.0701	290.0790	85.64	318.48	3658.05	48.51	628.23
**18**	Procyanidin B2	$C_{30}H_{26}O_{12}$	0.26	289.0701	578.1424	85.64	318.48	3658.05		628.23
**19**	Kaempferide	$C_{16}H_{11}O_6$	-0.48	299.0538	300.0633	65.28			156.25	64.35
**20**	Quercetin	$C_{15}H_9O_7$	-0.84	301.0340	302.0426	416.78		2524.55		
**21**	Taxifolin	$C_{15}H_{11}O_7$	-0.11	303.0499	304.0583	84.44				

N°	Compound	Molecular formula	Error (ppm)	Measured mass $[M-H]^-$	Monoisotopic mass	C-Residue	A-Residue	P-Residue	D-Residue	Pe-Residue
**22**	(Epi)gallocatechin	$C_{15}H_{13}O_7$	-3.29	305.0652	306.0739	109.97		711.45	62	
**23**	Vanillic acid-hexoside	$C_{18}H_{33}O_5$	-4.02	329.2318	330.0950	73.16	63.7		75.19	43.97
**24**	Coumaroylquinic acid	$C_{16}H_{17}O_8$	-0.35	337.0915	338.1001				39.45	
**25**	Retusin	$C_{19}H_{17}O_7$	0.38	357.0972	358.1052			2813.35		122.89
**26**	Feruloylquinic acid	$C_{17}H_{19}O_9$	0.82	367.1018	368.1107					54.15
**27**	Sinapoylglucose	$C_{17}H_{21}O_{10}$	-0.18	385.1119	386.1212		42.09			47.99

Table S6. Fold change values of the mobility of *C. elegans* treated with the different extracts at the three doses.

	Fold change mobility			
	**Day 1**	**Day 2**	**Day 3**	**Day 4**
Conventional (10µl/mL)	1.5	1.7	1.5	1.3
Conventional (20µl/mL)	1.2	1.3	1.3	1.3
Conventional (30µl/mL)	1.8	2.0	1.8	1.8
Alkaline (10µl/mL)	1.4	1.6	1.6	1.7
Alkaline (20µl/mL)	1.5	1.6	1.6	1.7
Alkaline (30µl/mL)	1.8	1.9	1.8	1.8
Acid (2.5µl/mL)	1.1	1.2	1.2	1.2
Acid (5µl/mL)	1.2	1.3	1.3	1.3
Acid (10µl/mL)	1.2	1.3	1.4	1.5
Promod HBN (10µl/mL)	1.0	1.1	1.3	1.2
Promod HBN (20µl/mL)	1.5	1.6	1.6	1.7
Promod HBN (30µl/mL)	1.5	1.7	1.7	1.6
Depol HBN (10µl/mL)	1.9	1.7	1.7	2.1
Depol HBN (20µl/mL)	1.5	1.4	1.2	1.6
Depol HBN (30µl/mL)	2.0	1.9	1.9	2.1
Pectinase HBN (10µl/mL)	1.7	1.8	1.6	1.6
Pectinase HBN (20µl/mL)	1.0	1.0	1.0	1.0

Pectinase HBN (30µl/mL)	1.2	1.2	1.3	1.3
Promod TPA (10µl/mL)	1.4	1.6	2.0	2.3
Promod TPA (20µl/mL)	1.3	1.6	2	2.3
Promod TPA (30µl/mL)	1.5	1.7	2.2	2.6
Promod TPA (30µl/mL)	1.5	1.7	2.2	2.6
Depol TPA (10µl/mL)	1.4	1.3	1.3	1.4
Depol TPA (20µl/mL)	1.1	1.1	1.2	1.5
Depol TPA (30µl/mL)	1.3	1.4	1.5	1.3
Pectinase TPA (10µl/mL)	2.0	2.0	1.8	1.5
Pectinase TPA (20µl/mL)	2.1	1.9	1.7	1.5
Pectinase TPA (30µl/mL)	2.1	1.7	1.7	1.6
Promod HB (10µl/mL)	1.2	1.3	1.5	1.9
Promod HB (20µl/mL)	1.1	1.2	1.6	2.1
Promod HB (30µl/mL)	1.1	1.2	1.6	2.1
Depol HB (10µl/mL)	0.9	1.0	1.3	1.7
Depol HB (20µl/mL)	1.0	1.2	1.3	1.4
Depol HB (30µl/mL)	1.2	1.3	1.5	1.7
Pectinase HB (10µl/mL)	1.8	2.1	2.0	1.6
Pectinase HB (20µl/mL)	1.6	1.8	1.7	1.4
Pectinase HB (30µl/mL)	1.3	1.5	1.6	1.3

Figure S1. UV absorption chromatograms at 280 nm with *Rf* values of cherry pomace (1: conventional extraction, 2: alkaline hydrolysis, 3: acid hydrolysis, 4: enzymatic hydrolysis by Depol enzyme, 5: enzymatic hydrolysis by Promod enzyme, 6: enzymatic hydrolysis by Pectinase enzyme).

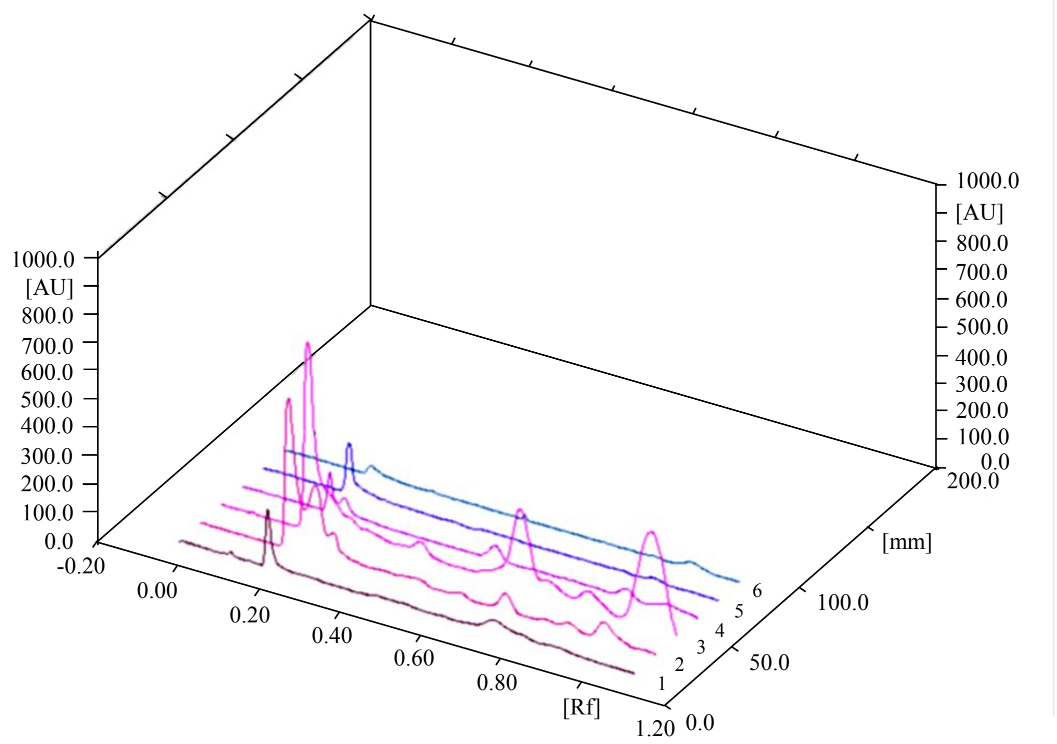

Figure S2. Effect of A) conventional, B) acid, C) alkaline, D) Pectinase HBN, E) Pectinase TPA, F) Pectinase HB, G) Promod HBN, H) Promod TPA, I) Promod HB, J) Depol HBN, K) Depol TPA and L) Depol HB extracts on body paralysis delay. *Gingko biloba* (Egb 761) was used as positive control. Data correspond to the average of two independent experiments.

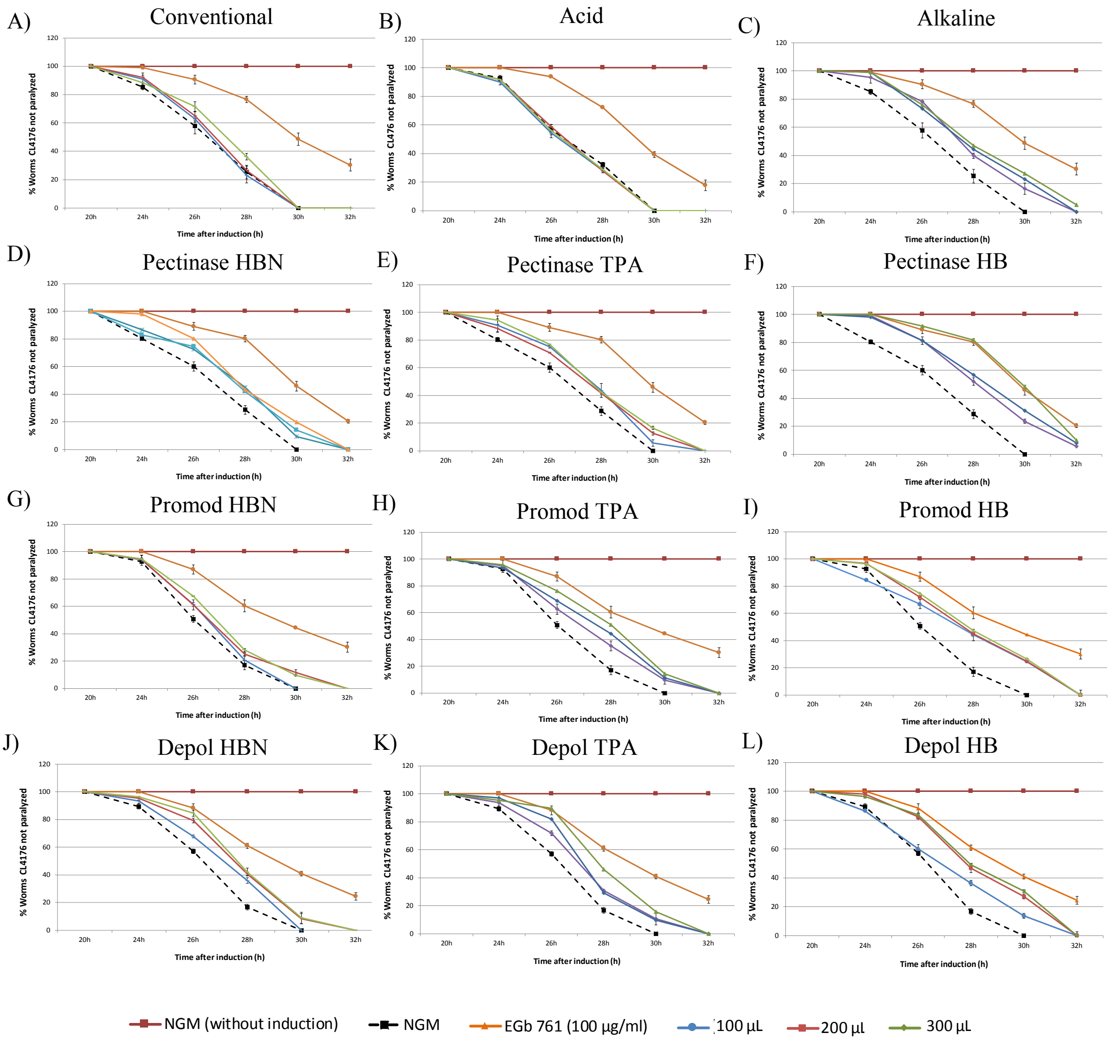

Supplement: Supplementary file 1 — jf2c03346_si_001.pdf [file jf2c03346_si_001.pdf]
